# Concordance of Microbiological and Histopathological Examination of Bone Biopsies in Diagnosing Diabetic Foot Osteomyelitis, a Systematic Review

**DOI:** 10.1002/jfa2.70223

**Published:** 2026-10-02

**Authors:** Levi Kadiri, Anthony Maher, Andy Bridgen

**Affiliations:** ^1^ University of Huddersfield Huddersfield UK; ^2^ Department of Podiatric Surgery County Health Partnerships, Park House Health and Social Care Centre Carlton Nottingham UK

**Keywords:** bone biopsy, diabetic foot osteomyelitis, diagnostic accuracy, diagnostic concordance, histopathology, microbiology

## Abstract

**Background:**

Diabetic foot osteomyelitis (DFO) is a serious complication of diabetic foot ulcers and is associated with increased risk of amputation, morbidity, and mortality. Bone biopsy remains the most definitive diagnostic modality, yet controversy persists over its diagnostic utility This study reviews the diagnostic concordance of histopathology and microbiology in bone biopsy specimens from patients with DFO and examines the clinical implications of the findings.

**Methods:**

A systematic review was conducted using PubMed, Medline, CINHAL, Cochrane Library, and Google Scholar. Studies included adult patients with DFO in whom bone biopsy specimens underwent both histopathological and microbiological analysis. Five core studies met the eligibility criteria. Data on study design, setting, population, diagnostic methods, concordance rates, and outcomes were extracted and synthesized utilising a quantitative narrative synthesis.

**Results:**

Across the five studies (*n* = 308 patients), histopathology and microbiology showed modest concordance, with mean rates of concordant positive results of 43.4% and common discordant findings. Histopathology demonstrated higher specificity, while microbiology had higher sensitivity, ranging from 70% to 83.9% but greater susceptibility to contamination. Positive histopathology was more strongly associated with the need for revision surgery or extended antibiotic therapy.

**Discussion:**

Histopathology appears to offer superior diagnostic specificity, whereas microbiology provides valuable information for antimicrobial management. Frequent discordance underscores the importance of interpreting results in a clinical context. Further robust, prospective studies are needed to clarify the optimal diagnostic approach and standardise definitions.

**Abbreviations**
API/BBL: Analytical Profile Index/Becton Dickinson Biochemical Laboratory (microbiology identification systems)
BLN assay: bone/lymph node assay, context‐dependent
CASP: Critical Appraisal Skills Programme
CT: computed tomography
DFO: diabetic foot osteomyelitis
DFU: diabetic foot ulcer
H&E: hematoxylin and eosin
IDSA: Infectious Diseases Society of America
IWGDF: International Working Group on the Diabetic Foot
LR+: positive likelihood ratio
LR−: negative likelihood ratio
MRI: magnetic resonance imaging
NICE: National Institute for Health and Care Excellence
NPV: negative predictive value
PPV: positive predictive value
PRISMA: preferred reporting items for systematic reviews and meta‐analyses
RCT: randomised controlled trial

## 1 Introduction

### 1.1 Background

Diabetes mellitus affects over 820 million people globally, with diabetic foot ulcers (DFUs) representing a major cause of morbidity, infection, and lower‐limb amputation [1]. Individuals with diabetes have an estimated 25% lifetime risk of developing foot ulcers, with infection present in a significant proportion [2]. Up to 60% of DFUs become infected, and among those, diabetic foot osteomyelitis (DFO) is a frequent and serious complication [3].

Approximately 15% of people with diabetes, are affected by osteomyelitis globally, and its prevalence in infected ulcers is as high as 66% [4, 5]. It results from contiguous spread from chronic ulcers rather than haematogenous dissemination [6]. Left untreated or misdiagnosed, osteomyelitis can lead to irreversible bone destruction, systemic infection, and limb loss [6], underscoring the importance of accurate and timely diagnosis [7].

Foot and ankle surgery is central to managing diabetic foot osteomyelitis (DFO), providing both diagnostic and therapeutic benefits. It enables targeted bone biopsy, debridement, and, when necessary, resection or minor amputation [8]. Accurate diagnosis is essential to guide the extent of bone removal and to prevent either undertreatment, or overtreatment [9].

A variety of diagnostic tools support clinical evaluation, including inflammatory markers, imaging modalities (radiography, MRI, CT), and the bedside probe‐to‐bone (PTB) test [10, 11]. While valuable, each has limitations in sensitivity, specificity, or availability, particularly when distinguishing soft tissue from bone infection [12]. Bone biopsy remains the most definitive diagnostic modality [13, 14] surgical bone biopsy has demonstrated superior diagnostic accuracy compared to PTB and imaging. In a longitudinal study of 1666 diabetic patients, the PTB test showed a moderate predictive value (0.57) and often required biopsy confirmation [15]. Similar findings have been reported in subsequent studies, emphasising that while PTB and imaging contribute to clinical suspicion, biopsy provides definitive confirmation [16, 17]. Also, a systematic review by Termaat et al. [18] found that advanced imaging such as FDG‐PET, though accurate, did not surpass biopsy in specificity and diagnostic certainty.

Bone biopsy specimens can undergo microbiological culture and histopathological examination [19]. Microbiology identifies causative pathogens to guide antimicrobial therapy but is susceptible to contamination and may misidentify colonisers as pathogens [20]. Histopathology, on the other hand, allows direct visualisation of inflammation and necrosis and is often considered the reference standard because it confirms infection within the marrow [21, 22]. Unlike microbiology and imaging, histopathology distinguishes acute from chronic osteomyelitis and is less affected by external contamination or reactive changes (Figure 1) [23, 24].

**FIGURE 1:**
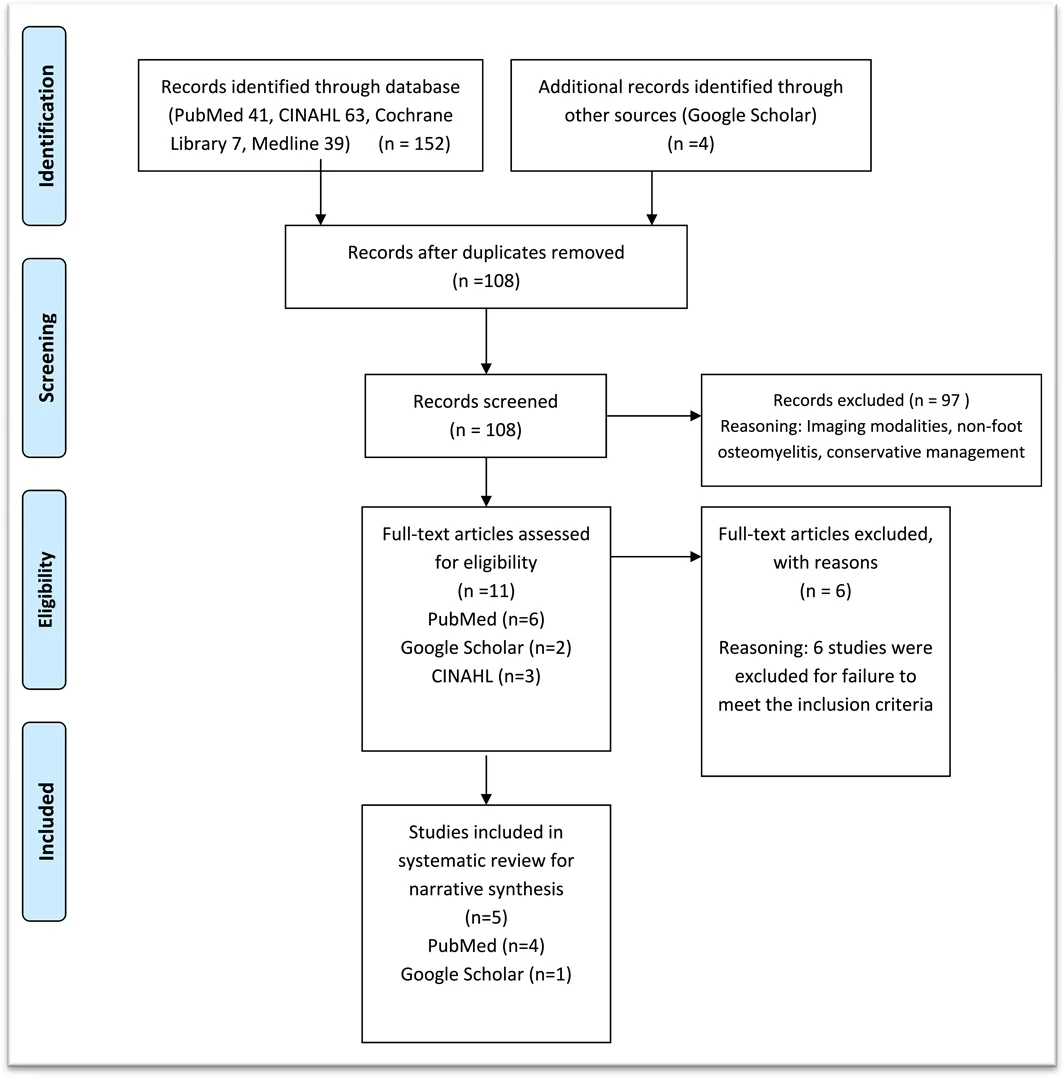
PRISMA flow chart of the selection process.

However, microbiology remains essential for antimicrobial guidance due to its quicker turnaround and lower cost [20, 23]. In clinical practice, microbiology is often prioritised, particularly where histopathology is delayed or unavailable, despite concerns over its diagnostic specificity [25, 26].

While histopathology is widely regarded as the diagnostic gold standard, it has practical limitations, including longer processing time and higher cost [27]. Discordance between microbiological and histopathological findings complicates clinical decisions, especially when one modality is positive and the other negative [28].

Current guidelines reflect these challenges. The Infectious Diseases Society of America (IDSA) and International Working Group on the Diabetic Foot (IWGDF) recommend bone biopsy for uncertain cases and advise that specimens be sent for both histopathology and microbiology to maximize diagnostic yield [29, 30]. Similarly, NICE guidelines acknowledge the superiority of biopsy but suggest selective use given its invasiveness and limited accessibility (NICE NG19 & CG119).

Despite advances in diagnostic modalities, there is a lack of high‐quality evidence clarifying the relative diagnostic accuracy, concordance, and clinical implications of histopathology versus microbiology. Ongoing studies, such as the DOMINO DFU multicentre cohort in the UK, aim to address this gap by evaluating diagnostic pathways and their impact on outcomes [31].

### 1.2 Aims and Objectives

This systematic review aims to critically evaluate and compare the diagnostic concordance and clinical utility of microbiological analysis and histopathological examination of bone biopsy specimens in the diagnosis of diabetic foot osteomyelitis (DFO). Specifically, the objectives are to assess the diagnostic accuracy between microbiological findings and histopathology across multiple studies, determine the degree of diagnostic concordance between these two investigative modalities, and identify gaps within the current literature to propose recommendations for future research and for the development of improved clinical protocols regarding bone biopsy analysis in DFO.

## 2 Methods

A systematic review was conducted to evaluate the diagnostic concordance and accuracy of histopathological and microbiological analysis of bone biopsy specimens in diabetic foot osteomyelitis (DFO). Electronic databases searched included PubMed, Medline, CINAHL, the Cochrane Library, and Google Scholar. The search covered articles published from January 2010 to June 2025 to reflect contemporary diagnostic standards and guidelines [29, 32]; (NICE 2019 [CG119 & NG19]) and the widespread adoption of multidisciplinary foot clinics in the UK.

Search terms were developed using the PICO framework (Population, Intervention, Comparator, Outcome), combining keywords, synonyms, and controlled vocabulary to ensure sensitivity and specificity. Boolean operators were employed to refine the results. The specific PICO terms are summarised in Table 1, while the detailed search strategy is provided in Table 2. Database‐specific strategies are listed in Supporting Information S1: Appendices 1–4.

**TABLE 1:** PICO search concept.

PICO element	Search terms
Population	“diabetic foot” OR “diabetic foot ulcer” OR “diabetic foot infection” OR “diabetic foot osteomyelitis” OR “diabetes‐related foot disease”
Intervention	“bone culture” OR “bone microbiology” OR “microbiological culture” OR “bacterial culture”
Comparison	“bone biopsy” OR “bone histology” OR “bone histopathology” OR “histopathological examination”
Outcome	“diagnostic accuracy” OR “sensitivity” OR “specificity” OR “predictive value” OR “diagnosis” OR “comparison” OR “concordance”

**TABLE 2:** Search strategy.

ID	Search
#1	exp Biopsy/
#2	“bone biopsy” OR “bone specimen” OR “bone tissue sample” OR “bone sampling” OR “osseous biopsy” OR “intraosseous biopsy”
#3	#1 OR #2
#4	exp Diabetic Foot/
#5	“diabetic foot” OR “diabetic foot ulcer” OR “DFU” OR “infected diabetic foot ulcer” OR “foot ulcer in diabetes” OR “neuropathic foot ulcer” OR “diabetic foot infection”
#6	#4 OR #5
#7	exp Osteomyelitis/
#8	“osteomyelitis” OR “bone infection” OR “infected bone” OR “diabetic foot osteomyelitis” OR “chronic osteomyelitis” OR “acute osteomyelitis” OR “osteitis” OR “infection of bone tissue”
#9	#7 OR #8
#10	exp histopathology/
#11	“histopathology” OR “histology” OR “histological analysis” OR “bone histology” OR “bone histopathology” OR “tissue analysis”
#12	#10 OR #11
#13	exp microbiology/
#14	“microbiology” OR “bone culture” OR “bacterial culture” OR “microbial analysis”
#15	#13 OR #14
#16	#12 OR #15
#17	#3 AND #6 AND #9 AND #16

Study selection was guided by the PICO framework [33]. Inclusion and exclusion criteria are detailed in Table 3. Briefly, studies were eligible if they met the following criteria:

**TABLE 3:** Eligibility criteria.

Study type & PICO	Inclusion	Exclusion
Study type	Peer‐reviewed and English language articles published between January 2010 and June 2025 Diagnostic accuracy studies, cross‐sectional studies, retrospective or prospective cohort studies, or case‐control studies comparing histopathology and microbiology in DFO Studies with at least 20 patients	Editorials, opinion pieces, and reviews without primary data Non‐human, animal/lab studies
Population	Adults (≥ 18 years) with diabetes mellitus presenting with diabetic foot ulcers (DFUs) and suspected or confirmed osteomyelitis	Studies on non‐diabetic patients, non‐foot ulcers, children, or trauma‐related osteomyelitis
Intervention	Studies involving bone biopsy followed by histopathological/microbiological analysis to diagnose diabetic foot osteomyelitis	Studies that only assessed soft tissue, swabs, or superficial cultures without a bone biopsy
Comparison	Microbiological/histopathological analysis of bone biopsy specimens from the same site or patient	Studies without comparative analysis between histopathology and microbiology (e.g., only one diagnostic modality used)
Outcome	Diagnostic accuracy measures such as sensitivity, specificity, positive predictive value, negative predictive value, or other valid performance indicators for histopathology and/or microbiology	No reporting of diagnostic accuracy or lack of detail on how histopathology/microbiology contributed to diagnosis

Population: Adults (≥ 18 years) with diabetes and diabetic foot ulcers (DFUs) undergoing bone biopsy for suspected or confirmed DFO.

Intervention: Bone biopsy analysed by both histopathology and microbiology.

Comparator: Comparative evaluation of histopathological and microbiological results from the same specimen or patient.

Outcomes: Diagnostic accuracy metrics (sensitivity, specificity, predictive values, concordance) or clinically relevant diagnostic performance measures.

Only peer‐reviewed, English‐language studies published between January 2010 and June 2025 were included. Eligible study designs included diagnostic accuracy studies, cross‐sectional, cohort, or case‐control designs with ≥ 20 participants to improve generalizability [34].

Studies were excluded if they lacked comparative analysis, were conducted in non‐diabetic or paediatric populations, focussed solely on soft tissue rather than bone biopsy, were reviews or editorials, or were non‐peer‐reviewed grey literature.

All search results were imported into a management software, where duplicates and non‐relevant records were removed. Single reviewer screened titles and abstracts, followed by full‐text assessment against predetermined eligibility criteria. Reasons for exclusion were documented, and the study selection process is presented in a PRISMA flow diagram [35]. Extracted data were organised into structured comparison tables, capturing study design, setting, participant characteristics, biopsy approach, laboratory methods, and diagnostic outcomes. Additional tables summarised quantitative performance metrics, including concordance and predictive values. Data extraction templates are provided in the appendices.

The methodological quality of the included studies was assessed using the Critical Appraisal Skills Programme (CASP) checklists [36]. Each study was categorised as having no concerns, minor concerns, or major concerns. Appraisal outcomes were summarised using a colour‐coded matrix to enhance transparency [37]. The CASP tool was selected because of its relevance to diagnostic and observational study designs and its established use within health research.

Risk of bias was assessed using the QUADAS‐2 tool. QUADAS‐2 was selected because it enables structured appraisal of patient selection, conduct, and interpretation of index tests, validity of the reference standard, and completeness of patient flow [38]. As a single reviewer conducted all assessments, the potential for subjectivity is acknowledged. However, two additional reviewers independently cross‐verified the review protocol, study selection process, and data extraction. Any disagreements were resolved through discussion, ensuring transparency and methodological rigour.

Primary outcomes included diagnostic sensitivity, specificity, positive and negative predictive values, overall diagnostic accuracy, and concordance between histopathology and microbiology. Secondary outcomes, where reported, included revision surgery rates and duration of antibiotic therapy. Owing to heterogeneity in study design, reference standards, and outcome definitions, a meta‐analysis was not appropriate. A quantitative narrative synthesis was therefore employed to summarise diagnostic performance across studies [39]. The certainty of evidence of this systematic review was assessed using the GRADE approach, which considers factors such as risk of bias, inconsistency, indirectness and imprecision [40].

## 3 Results

The initial search yielded 156 articles across MEDLINE, Google Scholar, PubMed, Cochrane, and CINAHL. Ultimately, 5 studies, published between 2010 and 2025, were included in the final synthesis. The complete selection process is illustrated in the PRISMA flowchart (Figure 1).

The 5 studies comprised two prospective cohorts, two retrospective cohorts, and one cross‐sectional study, conducted in Spain, Costa Rica, the USA, and Australia. Together, they included 308 patients (315 bone samples) with suspected or confirmed DFO. Sample sizes ranged from 35 to 93 patients, mean age ranged 44–69 years, and 71% of participants were male.

All studies compared the histopathology and microbiology of bone biopsies, collected intraoperatively during surgery or amputation.

Histopathology was treated as the reference standard in 4 studies, while one [41] used a composite reference including genetic sequencing.

Microbiological sensitivity ranged from 70% to 83.9%, specificity 25.8%–42%, and positive predictive values (PPV) were generally high (69%–92%). Negative predictive values (NPV) were consistently low (13%–44%).

Concordance between modalities varied substantially: positive concordance (both positive) ranged from 6.5% to 63.5% (mean: ∼45%), while discordance was common (Histo+/Micro−: mean 11%; Histo−/Micro+: mean 25%).

Further details on study settings, methods, diagnostic accuracy, and patient demographics are summarised in Tables 4, 5, 6, with full PICO data in Supporting Information S1: Appendix 5 and reasons for exclusion at full text analysis in Supporting Information S1: Appendix 7

**TABLE 4:** Characteristics of each study part B.

Author, year	Study design	Setting	Population	Sample size	Diagnostic methods compared	Specimen source	Outcome measures
Tardáguila‐García et al. [42]	Cross‐sectional study	Diabetic Foot Unit, Universidad Complutense de Madrid, Spain	Adults with diabetes, forefoot ulcers, and clinical suspicion of DFO	52 patients (from an initial 116 screened)	Bone biopsy: Histology versus Microbiology	Bone samples from surgical procedures	Sensitivity, specificity, PPV, NPV, likelihood ratios (bone culture vs. histology as gold standard)
Weiner et al. [23]	Matched case‐control study	Tertiary care teaching hospital, Midwestern USA	Adults with diabetes undergoing foot surgery for suspected infection	44 (specimens from 37 patients)	Bone biopsy split for: Histology Microbiology (aerobic, anaerobic, fungal culture)	Surgical debridement site (same sample hemisected for both tests)	Sensitivity, specificity, PPV, NPV, diagnostic accuracy, McNemar's test for significance
Víquez‐Molina et al. [43]	Prospective cohort study	Diabetic Foot Unit, San Juan de Dios Hospital, Costa Rica	Patients with diabetic foot osteomyelitis undergoing conservative surgery (non‐amputation resections)	93 patients	Histopathology versus microbiology on bone resection margins	Bone resection margins (proximal, macroscopical)	Residual osteomyelitis (per histopathology), microbiological contamination, PPV, NPV, sensitivity/specificity
Lavery et al. [41]	Prospective study	University of Texas Southwestern Medical Center, USA	Adults with diabetes and moderate to severe foot infections suspicious for DFO	35 patients	Traditional bone culture Bone histopathology Genetic sequencing (16S rRNA) Combined approaches	Bone biopsy samples (surgical)	Diagnostic incidence of DFO by method, operational definition impact, and overlap in test results
Jin et al. [26]	Retrospective cohort study	Tertiary institution, Canberra, Australia	Adults with diabetes and forefoot DFO or DFI undergoing minor amputation	84 patients included (after exclusions)	Histopathology versus microbiology on proximal bone chip from amputation site	Proximal bone from amputation margin (surgically collected)	Revision operation, positive microbiology, positive histopathology, antibiotic therapy, wound healing

**TABLE 5:** Characteristics of each study part B.

Study	Statistical methods	Findings
Tardáguila‐García et al. [42]	McNemar test; chi‐square; ORs; LR+/LR−; SPSS and Epidat used for analysis	Sensitivity (microbiology): 70% — Specificity: 40% — PPV: 92% — NPV: 13% — Histology confirmed 90.4% of cases
Weiner et al. [23]	McNemar's test, contingency tables, standard diagnostic metrics (Sn, Sp, etc.)	Sensitivity (microbiology): 75% — Specificity: 42% — PPV: 77% — NPV: 39% — Diagnostic accuracy: 66% — No significant difference between methods (*p* = 0.7963)
Víquez‐Molina et al. [43]	Chi‐square, Fisher's exact test, *t*‐test, Mann‐Whitney U; diagnostic accuracy metrics calculated	Microbiology: High sensitivity (83.9%), low specificity (25.8%) — PPV: 69.3%, NPV: 44.4% — *Pseudomonas aeruginosa* significantly associated with contamination
Lavery et al. [41]	Chi‐square; ORs; ANOVA; confidence intervals; comparisons of sensitivity across definitions	DFO incidence: 45.7% (histology), 68.6% (traditional culture), 82.9% (genetic sequencing) — All histology‐positive cases were also culture‐positive — Genetic sequencing found no unique cases missed by histology or culture

**TABLE 6:** Characteristics of each study part C concordance in diagnosis between Histopathology and Microbiology across studies *DFO‐ Diabetic foot osteomyelitis.

Outcome	Histopathology +	Microbiology +	Concordant + (both +)	Histo+ and Micro−	Histo− and Micro +	Concordant −(both −)	Study sample size with DFO
Tardáguila‐García et al. [42]	90.4% (47 cases)	69.2% (36 cases)	63.5% (33 cases)	26.9% (14 cases)	5.8% (3 cases)	3.8% (2 cases)	52
Weiner et al. [23]	72.7% (32 cases)	70.4% (31 cases)	54.5% (24 cases)	18.2% (8 cases)	15.9% (7 cases)	11.3% (5 cases)	44
Víquez‐Molina et al. [43]	66.7% (62 cases)	80.6% (75 cases)	55.9% (52 cases)	10.8% (10 cases)	24.7% (23 cases)	8.6% (8 cases)	93
Lavery et al. [41]	45.7% (16 cases)	68.6% (24 cases)	45.7% (16 cases)	0% (0 cases)	22.9% (8 cases)	31.4% (11 cases)	35
Jin et al. [26]	6.5% (5 cases)	66.2% (51 cases)	6.5% (5 cases)	0% (0 cases)	59.7% (46 cases)	33.8% (26 cases)	77
Mean % Outcomes^a^	56%	71%	45.23%	11.17%	25.80%	17.80%	

Abbreviations: Histo, histopathology; Micro, microbiology.

^a^ Mean: unweighted, descriptive.

The five included studies showed broadly similar biopsy methodologies, with all obtaining intraoperative bone samples, most commonly from forefoot bones or resection margins. Only Lavery et al. [41] also used percutaneous biopsy; the others relied solely on operative sampling. Histopathological protocols were consistent across studies, all using H&E staining with standard fixation, decalcification, and paraffin embedding. Several studies applied predefined criteria to classify acute and chronic osteomyelitis, and all histological assessments were performed by a single pathologist. Microbiological methods showed greater variability. While aerobic cultures were universal, only one study included anaerobic and fungal cultures. Sample processing techniques differed, ranging from mechanical homogenisation to direct plating, and only Lavery et al. [41] incorporated molecular diagnostics (16S rRNA sequencing). Blinding and specimen Matching were inconsistently reported. Most studies used histopathology as the reference standard, although one applied a composite diagnostic criterion Table 7.

**TABLE 7:** Biopsy methodology and diagnostic workflow.

Study	Sample site	Timing	Biopsy type	Histopathology protocol	Microbiology protocol	Blinding?	Matching?	Reference protocol
Tardáguila‐García et al. [42]	Forefoot bones (phalanx, metatarsal, sesamoid)	Intraoperative	Surgical (via conservative bone resection)	Formalin fixation, decalcification, paraffin embedding, H&E staining; evaluated by same pathologist; DFO classified as acute/chronic per criteria	Mechanical homogenization, aerobic culture (no anaerobes); ID via API/BBL systems; monomicrobial versus polymicrobial classification	Yes	No	Histopathology (as gold standard)
Weiner et al. [23]	Infected pedal bone	Intraoperative	Surgical debridement	H&E stain, diagnosis based on PMNs (acute) or lymphoplasmacytic infiltrates (chronic); pathology assessed OM via inflammation presence	Aerobic, anaerobic, and fungal cultures from split specimen; processed per standard microbiologic practice	Not specified	Yes	Histopathology (used for accuracy stats)
Víquez‐Molina et al. [43]	Proximal bone resection margin	Intraoperative	Surgical resection margin sample	Evaluated by single pathologist using predefined criteria for DFO	Aerobic culture only; qualitative assessment of bacterial growth	Not stated	No	Histopathology
Lavery et al. [41]	Surgical (foot bones)	Intraoperative or percutaneous	Surgical needle or biopsy	Routine formalin fixation, paraffin embeds, H&E stain	Aerobi culture; pathogen‐directed sensitivity analysis	No	No	Histology, culture, and/or sequencing
Jin et al. [26]	Proximal bone margin	Intraoperative	Surgical chip	Fragmented, decalcified, formalin‐fixed; standard microscopy	Aseptic culture on agar plates + enrichment broth	No	No	Histopathology (interpreted as gold standard)

The methodological quality of all five included studies was assessed using the Critical Appraisal Skills Programme (CASP) checklists appropriate for their respective designs. Three studies were rated as having minor methodological concerns, while the remaining two were classified as having major concerns. None of the studies achieved a rating of “no concerns,”. Results are summarised in Table 8a and 8b,with a completed example in Supporting Information S1: Appendix 6.

**TABLE 8a:** CASP studies checklist.

	Q1	Q2	Q3	Q4	Q5	Q6	Q7	Q8	Q9	Q10	Q11	Overall assessment
CASP diagnostic studies checklist												
Tardáguila‐García et al. [42]	Yes	Yes	Yes	Uncertain	Yes	Yes	Yes	Yes	Yes	Yes	Yes	Minor concerns
Lavery et al. [41]	Yes	No	Yes	Uncertain	Yes	Yes	Yes	Uncertain	Yes	Uncertain	Yes	Major concerns
Víquez‐Molina et al. [43]	Yes	Yes	Yes	Uncertain	Yes	Yes	Yes	Yes	Yes	Yes	Yes	Minor concerns
CASP cohort studies checklist												
Jin et al. [26]	Yes	Yes	Yes	Yes	No	Yes	Yes	Yes	Yes	Yes	Yes	Minor concerns
CASP case‐control study checklist												
Weiner et al. [23]	Yes	Yes	Yes	Yes	Yes	No	Uncertain	Uncertain	Yes	Yes	Yes	Major concerns

*Note:* No concerns = all responses are “Yes”. Minor concerns = most responses are “Yes”, with one “No” or “Uncertain” response. Major concerns = more than one response is rated “No” or “Uncertain”. Green shading indicates “Yes” (criterion met); red shading indicates “No” (criterion not met) or “Major concerns” in the overall assessment yellow shading indicates “Uncertain” or “Minor concerns” in the overall assessment.

**TABLE 8b:** Adapted CASP studies checklist questions.

CASP diagnostic studies checklist questions
1. Did the study address a clearly formulated research question? 2. Was there a comparison with an appropriate reference standard? 3. Did all patients get the diagnostic test and reference standard? 4. Are the results of the test free from being influenced by the results of the reference standard? 5. Is the disease status of the tested population clearly described? 6. Were the methods for performing the test described in sufficient detail? 7. What are the results? 8. How sure are we about the results? Consequences and costs of the alternatives performed? 9. Can the results be applied to your patients/the population of interest? 10. Can the test be applied to your patient or population of interest? 11. Were all outcomes important to the individual or population considered?
CASP cohort studies checklist questions
1. Did the study address a clearly focussed issue? 2. Was the cohort recruited in an acceptable way? 3. Was the exposure accurately measured to minimise bias? 4. Was the outcome accurately measured to minimise bias? 5. Have the authors identified all important confounding factors? 6. Have they accounted for the confounding factors in the design and/or analysis? 7. Was the follow‐up of subjects complete enough? 8. Was the follow‐up of subjects long enough? 9. Do you believe the results? 10. Can the results be applied to the local population? 11. Do the results of this study fit with other available evidence?
CASP case‐control study checklist questions
1. Did the study address a clearly focussed issue? 2. Did the authors use an appropriate method to answer their question? 3. Were the cases recruited in an acceptable way? 4. Were the controls selected in an acceptable way? 5. Was the exposure accurately measured to minimise bias? 6. Have the authors taken account of the potential confounding factors in the design and/or in their analysis? 7. Was the treatment effect large? 8. Was the estimate of the treatment effect precise? 9. Do you believe the results? 10. Can the results be applied to your patients/the population of interest? 11. Do the results of this study fit with other available evidence?

Bias within the extracted studies was assessed utilising the QUADAS‐2 tool and demonstrated generally low concerns in patient selection and flow/timing, but unclear risk of bias in the conduct and interpretation of the index test and reference standard across all included studies as seen in Table 8c, with a completed example in Supporting Information S1: Appendix 8.

**TABLE 8c:** Condensed QUDAS‐2 table for risk of bias in QUDAS domains 1–4.

Study	D1: Patient selection	D2: Index test	D3: Reference standard	D4: Flow and timing
Lavery et al. [41]	Low	Unclear	Unclear	Low
Tardáguila‐García et al. [42]	Low	Unclear	Unclear	Low
Weiner et al. [23]	Low	Unclear	Unclear	Low
Jin et al. [26]	Low	Unclear	Unclear	Low
Víquez‐Molina et al. [43]	Low	Unclear	Unclear	Low

The QUADAS‐2 assessment suggests that methodological limitations were mainly related to inadequate reporting of blinding rather than fundamental design flaws. Although patient selection, sampling processes, and complete follow‐up were strong across studies, the uncertain risk of bias in the interpretation of both the index test and reference standard may contribute to variability in reported diagnostic agreement. These limitations should be considered when interpreting the comparative diagnostic accuracy of microbiology and histopathology in diabetic foot osteomyelitis.

Jin et al. [26] evaluated clinical outcomes (revision surgery, antibiotic extension). When both tests were positive, revision surgery occurred sooner and more often (80% vs. 19.6% for Micro^+^ alone), with higher rates of extended antibiotics. These findings suggest greater clinical trust in histopathology (Table 9).

**TABLE 9:** Clinical utility: Revision surgery and antibiotic extension as outcome measures key diagnostic performance [26].

Diagnostic metric	Positive microbiology only	Positive histopathology
Rate in diabetic foot osteomyelitis case	66.2% (51/77)	6.5% (5/77)
Revision surgery	25.5%	80%
Mean revision time	60.6 days	18.8 days
Antibiotic extension	8.7%	20%

All studies reported substantial discordance between histopathology and microbiology: Concordant positive (both+): 6.5%–63.5% (mean 45%), Histo+/Micro–: 0%–26.9% (mean 11%), Histo–/Micro+: 5.8%–59.7% (mean 25%). These patterns underscore the diagnostic challenges of relying on a single modality (Table 10).

**TABLE 10:** Clinically relevant diagnostic patterns: Concordant +, Histo+ & Micro, and Histo− & Micro +.

Outcome	Concordant + (both +)	Histo+ and Micro−	Histo− and Micro +	Study sample size with DFO
Tardáguila‐García et al. [42]	63.5% (33 cases)	26.9% (14 cases)	5.8% (3 cases)	52
Weiner et al. [23]	54.5% (24 cases)	18.2% (8 cases)	15.9% (7 cases)	44
Víquez‐Molina et al. [43]	55.9% (52 cases)	10.8% (10 cases)	24.7% (23 cases)	93
Lavery et al. [41]	45.7% (16 cases)	0% (0 cases)	22.9% (8 cases)	35
Jin et al. [26]	6.5% (5 cases)	0% (0 cases)	59.7% (46 cases)	77
Mean % Outcomes^a^	45.23%	11.17%	25.80%	

^a^ Mean: unweighted, descriptive.

Three studies reported diagnostic accuracy metrics using histopathology as the reference standard. Microbiology showed good sensitivity (70%–84%, mean 76%) but poor specificity (26%–42%, mean 36%). PPV was consistently high (69%–92%), whereas NPV was low (13%–44%). The overall diagnostic accuracy was modest (64%–66%). Table 11 summarises these measures.

**TABLE 11:** Diagnostic validity measures: Sensitivity, specificity, and predictive values.

Predictive values, validity measures and likelihood ratios									
Study	Diagnostic tool	Sensitivity (%)	Specificity (%)	PPV (%)	NPV (%)	LR+	LR‐	Study‐summised accuracy	Notes
Tardáguila‐García et al. [42]	Bone culture (v Histo)	70	40	92	13	1.20	0.86	Not specified	Histopathology was used as the reference standard. McNemar test showed a significant difference between tests (*p* = 0.013)
Weiner et al. [23]	Microbiology (v Histo)	75	42	77	39	0.53	0.60	66%	Histopathology used as reference standard. No statistically significant difference between tests (McNemar *p* = 0.7963)
Víquez‐Molina et al. [43]	Microbiology (v Histo)	83.9	25.8	69.3	44.3	—	—	64.5%	Histopathology was considered the gold standard for residual osteomyelitis
Mean specificity sensitivity^a^		76.3	35.9						

^a^ Mean: unweighted, descriptive.

## 4 Discussion

This review evaluated the comparative diagnostic performance, concordance, and clinical utility of histopathological and microbiological analysis in diabetic foot osteomyelitis (DFO). Across five core studies [23, 26, 41, 42, 43], microbiology typically demonstrated higher sensitivity, whereas histopathology produced greater specificity and stronger associations with clinical outcomes. Concordance between modalities was suboptimal, reinforcing the need for multimodal diagnostic strategies rather than reliance on a single test.

The included studies differed in the clinical purpose and context of bone sampling (Table 7). Weiner et al. [23] and Tardáguila‐García et al. [42] examined bone suspected of osteomyelitis obtained during surgical debridement or conservative resection, with specimens divided for histopathological and microbiological assessment. Within this context, Weiner et al. reported relatively balanced discordance between the two modalities, whereas Tardáguila‐García et al. predominantly identified histopathology‐positive/microbiology‐negative findings, particularly in chronic osteomyelitis. In contrast, Víquez‐Molina et al. [43] and Jin et al. [26] assessed proximal bone margins following conservative surgery and minor amputation, respectively, and reported more frequent microbiology‐positive/histopathology‐negative findings than the reverse pattern. These margin studies evaluated residual infection after resection rather than the diagnosis of infection in the removed bone. Lavery et al. [41] included both percutaneous and intraoperative sampling but did not report diagnostic findings separately by route, limiting direct comparison with either sampling context. Differences in specimen selection, resection extent, laboratory processing and diagnostic criteria further restrict comparability. These patterns therefore require context‐specific interpretation and cannot establish that sampling method alone explains the observed discordance. The considerable heterogeneity in microbiological techniques, particularly the inconsistent inclusion of anaerobic and fungal cultures, as well as the limited use of molecular diagnostics, likely contributes to variability in diagnostic accuracy across studies. The inconsistent reporting of blinding and specimen matching further affected internal validity (Table 7). While histopathology was commonly used as the reference standard, the use of composite criteria in one study highlights the evolving nature of diagnostic approaches. Collectively, these variations reduce comparability between studies and emphasise the need for more standardised and reproducible diagnostic workflows in future DFO research.

When compared using diagnostic predictive values, microbiology demonstrated higher sensitivity (mean 76.3%) but poor specificity (mean 35.9%), with high PPVs (up to 92%) but consistently low NPVs (13%–44%) (Table 11). These findings, consistent with earlier studies [23, 44], suggest that microbiology alone may risk overdiagnosis and overtreatment, particularly where contamination or colonisation influences culture positivity. Histopathology demonstrated greater specificity (reported up to 94%) and appeared less susceptible to contamination, supporting its role as a more reliable confirmatory investigation [45, 46]. Variability in histopathology positivity rates (6.5%–90.4%) likely reflects differences in sampling technique, diagnostic thresholds, disease chronicity, and observer interpretation. In addition to these quantitative performance characteristics, it is important to recognise that diabetes‐related chronic inflammation itself may confound histopathological interpretation, particularly in early or borderline cases of osteomyelitis [22].

Histopathology and microbiology demonstrated only moderate agreement, with mean concordant‐positive rates around 45% (Tables 6 and 10). Discordance was common, particularly in cases with microbiology‐positive/histopathology‐negative discordance, possibly representing contamination, colonisation, or superficial infection rather than true osteomyelitis [26, 47, 48]. These findings align with literature demonstrating that culture positivity does not always reflect active bone infection and may lead to overtreatment [49, 50]. Although contamination during sampling is one possible explanation, discordance may also reflect differences in the tissue submitted for each test, heterogeneous distribution of infection, specimen processing, or limitations in histopathological sensitivity and interpretation. Conversely, Discordant histopathology‐positive/microbiology‐negative findings may reflect failure to recover viable organisms, including the potential effects of preceding antibiotic exposure and culture conditions. These cases were less frequent but remain clinically important as they raise concern for underdiagnosis where viable organisms cannot be cultured [51, 52]. IWGDF [30] and IDSA [29] guidelines acknowledge the possibility of false‐positive residual bone cultures, while also recognising limitations in histopathological reproducibility and conflicting evidence regarding agreement between the methods.

Interpretation of diagnostic findings in diabetic foot osteomyelitis requires careful clinical correlation, particularly when microbiological and histopathological findings are discordant [53]. Although positive bone cultures may indicate genuine osteomyelitis, they may also reflect contamination during specimen collection or colonisation from adjacent soft tissue and chronic ulcer environments [53]. This distinction is clinically important because diabetic foot ulcers are frequently polymicrobial and heavily colonised, increasing the risk of false‐positive microbiological findings if culture results are interpreted in isolation [54]. Within the studies included in this review, several cases demonstrated positive microbiology despite negative histopathology, suggesting that microbiological positivity alone does not necessarily confirm active bone infection [26, 55]. This finding is supported by Mijuskovic et al. [56], who demonstrated that positive residual bone cultures substantially exceeded histological confirmation following toe and forefoot amputation, suggesting that microbiology may overestimate true osteomyelitis due to contamination of intraoperative samples. However, microbiology‐positive/histopathology‐negative findings alone do not establish contamination. Víquez‐Molina et al. [43] defined microbiology‐positive/histopathology‐negative specimens as false‐positive cultures and assumed that this represented contamination. This interpretation depended on the use of histopathology as the reference standard rather than independent confirmation of contamination. Jin et al. [26] likewise found frequent positive margin cultures without histological confirmation, but some of these cases subsequently required revision surgery, indicating that such findings cannot uniformly be dismissed as clinically irrelevant.

The clinical significance of a positive culture depends partly on the virulence and pathogenicity of the isolated organism [57]. Isolation of highly virulent organisms, particularly *Staphylococcus aureus*, is generally considered more indicative of true osteomyelitis and has consistently been associated with clinically significant bone infection in the DFO literature [58]. Interpretation becomes much more complex when low‐virulence organisms such as coagulase‐negative staphylococci (CoNS) are isolated [59]. Traditionally regarded as skin commensals or contaminants, increasing evidence suggests that certain CoNS species, particularly *Staphylococcus* epidermidis, may act as clinically significant pathogens in osteomyelitis, especially in diabetic or immunocompromised populations [60, 61]. Armstrong et al. [58] demonstrated frequent isolation of CoNS from deep bone cultures in diabetic foot infection, many of which displayed methicillin resistance, supporting the possibility that these organisms may represent true pathogens in selected clinical contexts. The proposed mechanisms include biofilm production and tissue adherence, facilitating persistent low‐grade infection and antimicrobial tolerance.

The debate regarding whether CoNS represent contaminants or true pathogens remains unresolved. Tattevin et al. [57] questioned the high proportion of CoNS isolated from bone biopsy specimens in the work of Senneville et al. [62]; however, Senneville and colleagues argued that repeated recovery of CoNS from carefully obtained deep bone specimens under strict operative conditions suggested genuine pathogenic significance rather than simple contamination. This interpretation is further supported by the observation that CoNS were isolated more frequently from bone biopsy specimens than from superficial swabs, implying that these organisms may not merely reflect surface colonisation [62]. Nevertheless, interpretation remains challenging when organisms are isolated from a single specimen or when the sampling route, aseptic precautions and specimen handling procedures are insufficiently described [57].

Positive histopathology demonstrated greater clinical relevance across the included studies. Histological evidence of inflammatory infiltration, marrow oedema, fibrosis, necrosis, or cortical destruction provides direct evidence of active bone infection and was more consistently associated with clinically significant outcomes, including revision surgery and prolonged antimicrobial therapy [26, 56]. In the retrospective study by Jin et al. [26], positive histopathological findings were significantly associated with revision surgery (80% vs. 25.5% for microbiology) and led to shorter times to reoperation (mean 18.8 vs. 60.6 days) and higher rates of extended antibiotic therapy (20% vs. 8.7%) (Table 9). Therefore, positive histology may represent a stronger indicator of clinically significant osteomyelitis requiring escalation of either surgical or medical management. However, the optimal duration of antibiotic therapy remains uncertain, as highlighted by Mijuskovic et al. [56], and further research is still required to clarify optimal treatment strategies.

In contrast, negative histopathology substantially reduces the likelihood of clinically significant osteomyelitis but should not be interpreted as absolute exclusion of infection [24]. Early disease, sampling error, limited biopsy volume, or prior antimicrobial exposure may contribute to false‐negative findings despite persistent infection elsewhere within the affected bone [63]. Consequently, histopathology‐negative/microbiology‐positive findings may represent contamination but may also reflect infection not identified in the tissue examined histologically. Neither explanation should be assumed solely from the discordant test combination; clinical findings and sampling conditions remain essential to interpretation [47, 48].

Prior antibiotic exposure represents an additional confounding factor influencing diagnostic interpretation [64]. Senneville et al. [62] highlighted the importance of obtaining bone biopsy following an antibiotic‐free interval where clinically feasible, as prior antimicrobial exposure may contribute to false‐negative culture results and diagnostic discordance. Histopathological assessment appears to be less directly influenced by preceding antibiotic therapy because inflammatory infiltrates and structural bone changes may persist despite temporary suppression of microbial growth [41]. Tardáguila‐García et al. [42] reported cessation of antibiotics for 48–72 hours before sampling, whereas most participants in Lavery et al. [41] received empirical antibiotics on admission. The other included studies did not consistently report detailed pre‐biopsy exposure and withholding protocols. Antibiotics may reduce microbiological recovery and contribute to histopathology‐positive/microbiology‐negative findings, although these studies cannot quantify their effect. The IWGDF/IDSA [30] guidelines acknowledge uncertainty regarding the optimal antibiotic‐free interval before bone biopsy. Antibiotic exposure should therefore be considered alongside sampling and laboratory factors, rather than assumed to explain all forms of discordance.

The method of specimen acquisition also substantially influences interpretation. Percutaneous bone biopsy aims to minimise contamination by accessing bone through intact skin and is considered microbiologically reliable when performed under sterile conditions [65, 66]. However, intraoperative sampling during amputation or debridement may be more susceptible to contamination from surrounding infected soft tissue despite careful aseptic technique [65]. Obtaining multiple concordant bone specimens may improve diagnostic reliability by increasing the reproducibility of microbiological findings and helping distinguish true pathogens from contaminant organisms, particularly in cases involving low‐virulence bacteria such as coagulase‐negative staphylococci [56, 60]. Taken together, these findings support the concept that histopathology and microbiology provide complementary rather than interchangeable diagnostic information in DFO. Reliance on microbiology alone risks overdiagnosis and unnecessary antimicrobial therapy, whereas histopathology alone cannot identify causative organisms or guide targeted antimicrobial treatment. Therefore, a multidisciplinary and context‐specific interpretation remains essential to differentiate true osteomyelitis from contamination or colonisation and to guide appropriate medical and surgical management.

When considering clinical utility and implications, histopathology demonstrated a stronger influence on clinical management decisions. Positive histological findings were associated with increased rates of revision surgery and prolonged antimicrobial therapy in multiple studies [26, 55]. Both Jin et al. [26] and Víquez‐Molina et al. [43] observed that positive histopathology was predictive of worse outcomes, whereas positive microbiology alone did not reliably predict poor clinical progression. These findings suggest that surgeons may regard histological confirmation as a more reliable indicator of true residual infection requiring further intervention. Histopathological evaluation therefore appears more closely aligned with clinical decision‐making than microbiology alone. In surgical settings, particularly where resection margins are assessed for residual infection, histopathology may provide the most reliable diagnostic anchor [24, 45].

Nonetheless, histopathology presents limitations that affect real‐time clinical decision‐making, including slower turnaround times, often exceeding 7 days due to specimen processing and decalcification requirements, compared with microbiological culture, which may provide results within 2 to 3 days [41, 67]. Inter‐observer variability may also reduce diagnostic reproducibility [68, 69]. For these reasons, microbiology remains essential for immediate postoperative care, accurate pathogen identification, and antibiotic susceptibility profiling. Even with lower specificity, microbiology provides actionable therapeutic guidance that histopathology alone cannot [44, 70].

In all, evidence from this review supports the following principles in Table 12.

**TABLE 12:** Diagnostic approach for clinical scenarios.

Clinical scenario	Best‐supported diagnostic approach
Assessing residual infection at resection margin	Histopathology as primary determinant
Rapid antimicrobial decision making	Initial microbiology results
High uncertainty or discordant findings	Combined interpretation with MDT input

Microbiology‐positive/histopathology‐negative findings (Table 10) raise concern about overtreatment when culture results are interpreted in isolation, but they do not independently establish that treatment is unnecessary [47]. Correlation with clinical severity, imaging, ulcer characteristics, and biopsy methodology remains important, particularly when assessing residual surgical margins [26, 71]. The IWGDF/IDSA [30] guidelines support aseptic bone sampling for culture, either intraoperatively or percutaneously, and acknowledge limitations in both microbiological and histopathological assessment. Therefore, discordant findings require interpretation alongside the clinical presentation and sampling context.

### 4.1 Future Directions

There remains a clear need for larger prospective, blinded studies using standardised histological and microbiological protocols. Future research should further explore the role of molecular diagnostics, algorithm‐driven histopathology, and artificial intelligence‐assisted interpretation in improving diagnostic yield and reducing observer bias. Longitudinal studies evaluating the impact of histology‐ or microbiology‐guided treatment decisions on clinically meaningful outcomes, including limb preservation, antimicrobial resistance, recurrence, and patient‐reported quality of life, are also required [72, 73]. Future research may additionally explore whether selective histopathological assessment based on microbiological profiles or suspected contaminant organisms could provide a cost‐effective alternative to routine combined testing, although current international guidelines continue to recommend multimodal diagnostic assessment. Emerging studies such as DOMINO‐DFU and novel therapeutic strategies including bacteriophage therapy may further influence future diagnostic interpretation and antimicrobial stewardship in DFO [4].

### 4.2 Strengths and Limitations

This review employed a structured comparative framework that enabled the synthesis of diagnostic accuracy and clinical relevance across varied clinical settings. The review also benefits from a combined sample size of 308 patients, strengthening the overall robustness of findings. However, the relatively small number of included studies demonstrates a continuing research gap within this field [74], while methodological heterogeneity limits the feasibility of conducting a quantitative meta‐analysis [75]. In addition, most included studies evaluated intraoperative or post‐amputation bone sampling rather than percutaneous biopsy techniques, limiting the generalisability of findings to non‐operative diagnostic settings where contamination risks and sampling characteristics may differ. Prior antibiotic exposure before bone biopsy was also inconsistently reported across the included studies, potentially influencing microbiological sensitivity and contributing to false‐negative culture results.

Furthermore, most studies were undertaken in surgical cohorts, restricting their applicability to non‐operative populations [76]. The frequent use of histopathology as the reference standard further assumes diagnostic infallibility and constrains an unbiased evaluation of microbiology [77]. Nevertheless, the emphasis on clinically meaningful outcomes strengthens the relevance of this review's conclusions for podiatric surgical practice.

The overall certainty of the evidence in this systematic review may be considered moderate when assessed using the GRADE approach [40]. This is primarily due to factors such as observational study designs, potential risk of bias, small sample sizes, and variability in diagnostic definitions and methodologies. As such, while the findings offer important insights into histopathology and microbiology concordance in diabetic foot osteomyelitis, they should be interpreted with appropriate caution. Refer to Supporting Information S1: Appendix 9 for table showing the full GRADE tool and assessment.

## 5 Conclusion

Histopathology provides important diagnostic information in DFO and may be particularly informative when assessing residual infection at surgical resection margins. Microbiology remains essential for pathogen identification and antimicrobial guidance. However, histopathology‐negative/microbiology‐positive findings should not automatically be classified as contamination, as their significance depends on sampling conditions, specimen processing, clinical context and the limitations of both diagnostic methods.

The findings of this review support complementary interpretation of histopathology and microbiology within a multidisciplinary assessment, with explicit consideration of differences between suspected infected bone, residual resection margins and percutaneous sampling.

Future research should aim to standardise diagnostic definitions and methodology, incorporate emerging molecular diagnostics, and focus on long‐term clinically meaningful outcomes in order to optimise the management of diabetic foot osteomyelitis. Future research may also explore whether selective histopathological assessment based on microbiological profiles or suspected contaminant organisms could provide a cost‐effective alternative to routine combined testing, although current international guidelines continue to recommend multimodal diagnostic assessment.

## Author Contributions

**Levi Kadiri:** conceptualization, methodology, investigation, data curation, formal analysis, visualization, project administration, writing – original draft, writing – review and editing. **Anthony Maher:** methodology, supervision, validation, writing – review and editing. **Andy Bridgen:** methodology, investigation, resources, supervision, validation, writing – review and editing.

## Funding

The authors have nothing to report.

## Ethics Statement

Ethical approval was granted by the University of Huddersfield Course Ethics Panel. As this systematic review used previously published data and involved no direct participation or identifiable personal information, individual consent to participate was not applicable.

## Conflicts of Interest

The authors declare no conflicts of interest.

## Supporting information


Supporting Information S1

Supporting Information S2

Supporting Information S3

## Data Availability

Data sharing not applicable to this article as no datasets were generated or analysed during the current study.
