# Supplementary material for: Concordance of Microbiological and Histopathological Examination of Bone Biopsies in Diagnosing Diabetic Foot Osteomyelitis, a Systematic Review
Source: J Foot Ankle Res. 2026 Oct 2;19(4):e70223. doi: 10.1002/jfa2.70223 (PMC13632924; doi:10.1002/jfa2.70223)
Supplement: Supplementary file 1 [file JFA2-19-e70223-s001.docx]

**Appendices**

Appendix

Appendix 1 EBSCO CINHAL search

Apendix 2 EBSCO Medline search

Appendix 3 Cochrane search

Appendix 4 Google scholar search

Appendix 5: PICO data extraction tool

| **Study Name** | **Are we misdiagnosing diabetic foot osteomyelitis? Is the gold standard gold?**  Lavery, L. A., Crisologo, P. A., La Fontaine, J., Bhavan, K., Oz, O. K., & Davis, K. E. (2019) |
| --- | --- |
| Participants | - 35 adult patients (≥21 years) with moderate to severe diabetic foot infection and clinical suspicion of osteomyelitis (DFO) - recruited prospectively from July to October 2019 at the University of Texas Southwestern Medical Center. Inclusion based on clinical signs (e.g., positive probe-to-bone test), radiographic/MRI evidence. |
| Intervention | Three diagnostic tests on bone biopsy specimens:   - Histopathology - Traditional microbiological culture (hospital microbiology lab) - Genetic sequencing (16S rRNA) (Pathogenius Lab).   Bone samples obtained via percutaneous biopsy or surgical debridement. |
| Comparators | Each diagnostic test was compared to others across multiple operational definitions:   - Histology vs. Traditional Culture - Histology vs. Genetic Sequencing - Traditional Culture vs. Genetic Sequencing - Combined modalities (histology + culture, histology + sequencing) |
| Outcomes | Primary: Incidence of osteomyelitis based on five operational definitions (each individual method and combined approaches)  - Key Result Summary:   - Histology only: 45.7% incidence - Traditional culture only: 68.6% incidence - Genetic sequencing only: 82.9% incidence - Histology + traditional culture: same as culture (68.6%) - Histology + sequencing: same as sequencing (82.9%)   - Histology did not detect any cases missed by culture or sequencing.  - Significant difference in detection: Genetic sequencing vs. histology (p = .002, OR 5.74, 95% CI 1.91–17.28).  - No significant difference: Genetic sequencing vs. traditional culture (p = .17).  - Clinical variables like skin perfusion pressure and ESR showed significance between groups |
| Study design | Prospective cohort pilot study; diagnostic comparison of three modalities applied to the same bone biopsy samples. Descriptive and inferential statistics were applied to operational definitions and demographic/clinical variables. |
| Key findings | Histology alone was the least sensitive and identified no additional cases beyond those picked up by culture/sequencing.  - No method was declared superior; each had limitations.  - Study highlighted variability in DFO diagnosis depending on which reference definition (test or combination) is used. |
| Conclusion | Diagnostic yield for DFO varies significantly based on the method used. Histology alone may miss cases, while genetic sequencing may over-identify due to inclusion of non-viable organisms. Combination of traditional culture and histology remains the standard, though genetic methods offer future promise. Larger studies are needed to define optimal strategies. |
| **Study Name** | **Histopathology is More Reliable Than Microbiology for Detecting Residual Osteomyelitis After Conservative Surgery for Diabetic Foot**  Víquez-Molina, G., Rojas-Bonilla, J. M., & Aragón-Sánchez, J. (2025) |
| Participants | - 93 adult patients with diabetes mellitus undergoing conservative surgery (bone resection preserving foot structure) for diabetic foot osteomyelitis (DFO) at a tertiary center in Costa Rica.   Subgroups included:   - OM1 (osteomyelitis without soft tissue infection), - OM2a (with deep abscess/cellulitis/necrosis) - OM2b (osteomyelitis diagnosed after wound healing from initial soft tissue infection). |
| Intervention | - Histopathological examination of bone resection margins (proximal to infected site). - Samples evaluated using predefined criteria by a single pathologist for diagnosis of residual osteomyelitis. Considered the gold standard for this study. |
| Comparators | - Microbiological culture of matching bone resection margin samples (aerobic only). - Contamination defined as positive culture with negative histology. Diagnostic metrics calculated using histology as the reference standard. |
| Outcome | - Microbiology performance at margins:    • Sensitivity: 83.9%    • Specificity: 25.8%    • PPV: 69.3%    • NPV: 44.4%    • Diagnostic Accuracy: 64.5%    - Concordance: 52 true positives, 8 true negatives, 23 false positives (contamination), 10 false negatives.  - Contamination Factors: Only *Pseudomonas aeruginosa* was significantly associated with contamination (p = .008); no association with polymicrobial infections, soft tissue infection, or severity of disease. |
| Study design | Prospective cohort study, nested within an earlier cohort. All patients underwent standardised conservative foot surgery by the same surgical team. Strict aseptic protocols were used, and matched margin samples were split for microbiology and histology. |
| Key findings | Microbiology showed high sensitivity but poor specificity at bone margins, leading to high false-positive rates (contamination).   - Histopathology was more reliable and not influenced by soft tissue infection or species diversity. - *Pseudomonas aeruginosa* uniquely associated with false-positive cultures at margins. - No significant correlation found between contamination and PAD, WBC, ESR, CRP, or HbA1c. - Polymicrobial infections not linked to increased contamination risk. |
| Conclusion | Histopathology should be prioritized over microbiology when evaluating residual osteomyelitis. Microbiological positivity often reflects contamination, not true infection—particularly when *P. aeruginosa* is present. Improved intraoperative protocols and possibly anti-biofilm strategies are recommended to reduce contamination and unnecessary antibiotic use. |
| **Study Name** | **Evaluation of routine proximal bone analysis for microbiology and histopathology in diabetes‐related foot infections and osteomyelitis.**  Jin, L., Chia, R. X., Neilson, W., Hardman, D., Frahm‐Jensen, G., & Jones, A. (2024) |
| Participants | - 84 adult patients with diabetes mellitus, undergoing minor amputations (transphalangeal or transmetatarsal) for suspected forefoot DFO at a tertiary hospital in Canberra, Australia (June–December 2020). - DFO diagnosis based on clinical, radiographic, CT, MRI, or nuclear scan. DFI patients had no preoperative evidence of osteomyelitis. |
| Intervention | - Histopathological examination of proximal bone margins obtained during amputation using sterile technique. - Specimens decalcified and examined microscopically for features of residual osteomyelitis. Used as the reference standard in this study. |
| Comparators | - Microbiological culture of the same proximal bone samples (aerobic culture only). - Cultures classified as positive or negative; positivity without histological confirmation was interpreted as likely contamination. |
| Outcome | Primary Outcomes:    • Concordance between microbiology and histopathology    • Need for revision surgery within 6 months    • Requirement for extended antibiotic therapy (>6 weeks)    - Diagnostic Performance (DFO cases):    • Histopathology positive in 5/77 (6.5%)    • Microbiology positive in 51/77 (66.2%)    • Histopathology + Microbiology: 5 cases    • Microbiology alone (false positive): 46 cases    • Sensitivity of microbiology: High (not quantified)    • Specificity: Poor; low positive predictive value    - Clinical Relevance:    • 80% of positive histology cases required revision (vs. 12.5% if histology negative, P = 0.01)    • Microbiology alone led to unnecessary treatment in some cases; most did well without revision/extended antibiotics    • Negative microbiology showed complete concordance with negative histology and no treatment failures |
| Study design | Single-centre retrospective cohort study with standardized surgical and sampling protocol. Data analysed via multivariable logistic regression. Histopathology used as the reference standard. Ethics-approved study with 6-month follow-up. |
| Key findings | - Histopathology was a stronger predictor of residual infection and need for revision than microbiology. - Microbiology had high sensitivity but low specificity, resulting in many false positives (likely contamination). - Pseudomonas aeruginosa identified in prior studies as a contamination risk, though not addressed here. - Higher pre-op CRP associated with both positive histology and revision (P = 0.02 and P = 0.01). - Mean histopathology reporting delay was 7.1 days, limiting its real-time impact. |
| Conclusion | Microbiology alone overestimates residual osteomyelitis due to likely contamination. Histopathology, while delayed, better correlates with clinical outcomes and should guide decisions on revision and antibiotic duration. Negative microbiology may be a reliable negative predictor, supporting shorter antibiotic courses and reduced need for aggressive intervention. |
| **Study Name** | **Histology versus microbiology for accuracy in identification of osteomyelitis in the diabetic foot.**  Weiner, R. D., Viselli, S. J., Fulkert, K. A., & Accetta, P. (2011). |
| Participants | - Adult patients with diabetes mellitus undergoing surgical intervention for suspected pedal osteomyelitis (OM) between January and September 2011 at a Midwestern U.S. tertiary care teaching hospital. - 44 specimens from 37 patients) - Clinical suspicion was based on clinical exam, standard radiographs, and in most cases MRI. |
| Intervention | Histopathological analysis of bone biopsy specimens obtained intraoperatively.   - Bone sections were fixed in 10% buffered formalin, decalcified, paraffin-embedded, HE-stained, and reviewed by pathologists. - Diagnosis of OM required presence of inflammation, with neutrophil predominance for acute OM, and lymphocytes/plasma cells for chronic OM. |
| Comparators | Microbiological culture of matching bone biopsy halves (aerobic, anaerobic, and fungal) using standard sterile processing. Positive results indicated presence of microbial growth consistent with infection. |
| Outcome | Primary Outcome: Diagnostic concordance between histology and microbiology for OM diagnosis.  - Secondary Outcomes: Sensitivity, specificity, PPV, NPV, LR+, LR−, and diagnostic accuracy of microbiologic culture using histology as the reference standard.  - Statistical Analysis: McNemar’s test (P = 0.7963) indicated no significant difference in diagnostic outcomes between the two modalities. |
| Study design | Matched case-control diagnostic study of 44 bone biopsy specimens from 37 diabetic patients. Each specimen was hemisected for both histologic and microbiologic testing. |
| Key findings | - Histology was positive in 72.7% of specimens; microbiology was positive in 70.4%.  - 75% of histology-positive cases were also microbiology-positive.  - Diagnostic accuracy of microbiology: 66%; sensitivity: 75%, specificity: 42%, PPV: 77%, NPV: 39%, LR+: 0.53, LR−: 0.60.  - McNemar’s test showed no statistically significant difference (P = 0.7963) between histology and microbiology in identifying OM. |
| Conclusion | In diabetic foot surgery, both histology and microbiology yield comparable diagnostic outcomes for OM. Although histology is considered the gold standard, the study suggests that microbiologic testing may be a cost-effective and sufficiently accurate alternative, especially given its ability to guide antibiotic therapy. Surgeon discretion in test choice is supported. |
| **Study Name** | **Diagnostic accuracy of bone culture versus biopsy in diabetic foot osteomyelitis.**  Tardaguila-Garcia, A., Sanz-Corbalan, I., Garcia-Morales, E., García-Álvarez, Y., Molines-Barroso, R. J., & Lazaro-Martinez, J. L. (2021). |
| Participants | - 52 adults with diabetes mellitus and clinically suspected diabetic foot osteomyelitis (DFO) based on positive probe-to-bone test and plain radiograph. - All patients had grade 3 diabetic foot ulcers per PEDIS classification, were undergoing conservative surgical intervention, and had forefoot ulcer location. |
| Intervention | - Bone biopsy with histopathological examination for diagnosis of osteomyelitis. - Samples fixed in 10% formalin, decalcified, embedded, and evaluated by a single pathologist blinded to clinical data. Histology categorised as acute or chronic osteomyelitis. |
| Comparators | - Bone biopsy with microbiological culture (aerobic only). Cultures assessed for bacterial growth, Gram stain classification (mono-/polymicrobial), and specific organisms identified using biochemical techniques. |
| Outcome | Diagnostic agreement between histopathology and microbiology.  - Sensitivity, specificity, positive predictive value (PPV), negative predictive value (NPV), and likelihood ratios (LR+, LR−) of bone culture compared to histology (gold standard).  - McNemar test for significance of diagnostic differences. |
| Study design | Cross-sectional diagnostic accuracy study of 52 patients (from initial 116 screened), conducted at a specialized diabetic foot unit in Spain |
| Key findings | Histopathology confirmed DFO in 90.4% of cases (acute: 25.5%; chronic: 74.5%).  - Bone culture sensitivity: 70%; specificity: 40%; PPV: 92%; NPV: 13%; LR+: 1.20; LR−: 0.86.  - Microbiology missed several chronic cases; all acute cases were culture positive.  - Statistically significant diagnostic discordance (P = 0.013). |
| Conclusion | Histology remains more reliable than microbiology for diagnosing DFO, particularly in chronic infections. Bone culture alone may underdiagnose due to false negatives. Combining both techniques is recommended when feasible. |

Appendix 6 completed CASP cohort tool

Appendix 7: Reasons for exclusion at full text analysis

| **Study** | **Title** | **Reason for exclusion at full text screening** |
| --- | --- | --- |
| Meyr et al., (2011) | Statistical reliability of bone biopsy for the diagnosis of osteomyelitis in the diabetic foot | Does not compare histopathology directly with microbiology or evaluate diagnostic accuracy metrics such as sensitivity or specificity. Instead, it focuses solely on interobserver agreement among pathologists |
| Gramberg et al., (2021) | Using a BonE BiOPsy (BeBoP) to determine the causative agent in persons with diabetes and foot osteomyelitis | As a protocol, it does not report any clinical or diagnostic accuracy outcomes, nor does it contain empirical results or comparative data between histopathology and microbiology |
| Elamurugan et al., (2011) | Role of bone biopsy specimen culture in the management of diabetic foot osteomyelitis | Does not assess or compare the diagnostic accuracy of microbiology versus histopathology, nor does it include histopathological analysis as an integral part of its methodology |
| Couturier et al., (2019) | Comparison of results obtained from per-wound bone biopsies versus transcutaneous bone biopsies in diabetic foot osteomyelitis | Does not compare histopathology and microbiology, nor does it report diagnostic accuracy metrics |
| Ulusoy et al., (2025) | Are Cultures a Reliable Alternative to Bone Biopsy for Microbiological Diagnosis of Diabetic Foot Osteomyelitis? | Focuses solely on microbiological comparison between deep tissue and bone biopsy cultures |
| Chadayammuri et al., (2017) | Diagnostic accuracy of various modalities relative to open bone biopsy for detection of long bone posttraumatic osteomyelitis. | Not specific to diabetic foot osteomyelitis, nor does it compare bone histopathology with bone microbiology |

Appendix 8: completed QUADAS-2 tool

Appendix 9: Grade analysis of Systematic review

**Review title**: Concordance of microbiological and histopathological examination of bone biopsies in diagnosing diabetic foot osteomyelitis, a systematic review

| Outcome | No. of Studies | Findings Summary | Certainty of Evidence (GRADE) | Reason for rating |
| --- | --- | --- | --- | --- |
| Diagnostic concordance between histopathology and microbiology | 5 | Concordance ranged from 6.5% to 55.9% across studies | ⬤⬤◯◯ (Low) | Downgraded for inconsistency (wide variation) and risk of bias (retrospective designs) |
| Specificity and sensitivity of microbiology using histopathology as reference | 4 | Microbiology had moderate sensitivity (70–83%) but low specificity (25–42% | ⬤⬤◯◯ (Low) | Downgraded for imprecision and inconsistency |
| Histopathology-positive but microbiology-negative results | 5 | Histo+/Micro− ranged from 0% to 26.9% | ⬤⬤◯◯ (Low) | Potential sampling bias; no standardised histological criteria |
| Impact on clinical decision-making (e.g. antibiotics, revision surgery) | 2 (Jin et al., Lavery et al.) | Histopathology associated with reoperation prediction; microbiology used more often for antimicrobial planning | ⬤⬤◯◯ (Low) | Indirectness and limited data on long-term outcomes |
| Use of both modalities together | 5 | Supplementary use improved diagnostic confidence; combined interpretation common in surgical settings | ⬤⬤⬤◯ (Moderate) | Consistent rationale, but studies lacked blinding or comparator arms |
