# Supplementary material for: Concordance of Microbiological and Histopathological Examination of Bone Biopsies in Diagnosing Diabetic Foot Osteomyelitis, a Systematic Review
Source: J Foot Ankle Res. 2026 Oct 2;19(4):e70223. doi: 10.1002/jfa2.70223 (PMC13632924; doi:10.1002/jfa2.70223)
Supplement: Supplementary file 2 [file JFA2-19-e70223-s002.docx]

CASP Checklist:

For diagnostic test studies

| **Reviewer Name:** | Levi Kadiri, Anthony Maher |
| --- | --- |
| **Paper Title:** | Diagnostic accuracy of bone culture versus biopsy in diabetic foot osteomyelitis |
| **Author:** | Tardaguila-Garcia et al |
| **Web Link:** | *DOI:* [10.1097/01.ASW.0000734376.32571.20](https://journals.lww.com/aswcjournal/abstract/2021/04000/diagnostic_accuracy_of_bone_culture_versus_biopsy.6.aspx?context=latestarticles) |
| **Appraisal Date:** | 09/01/2026 |

During critical appraisal, never make assumptions about what the researchers have done. If it is not possible to tell, use the “Can’t tell” response box. If you can’t tell, at best it means the researchers have not been explicit or transparent, but at worst it could mean the researchers have not undertaken a particular task or process. Once you’ve finished the critical appraisal, if there are a large number of “Can’t tell” responses, consider whether the findings of the study are trustworthy and interpret the results with caution.

| **Section A: Are the results of the study valid?** | | |
| --- | --- | --- |
| 1. Did the study address a clearly formulated research question? | ☐Yes ☐ No ☐ Can’t Tell  **X** | |
| *CONSIDER:*  *A question should include information about*   - *the population* - *the test* - *the setting* - *the outcomes* | | |
| 2. Was there a comparison with an appropriate reference standard? | ☐Yes ☐ No ☐ Can’t Tell  **X** | |
| *CONSIDER:*   - *Is this reference test(s) the best available indicator in the circumstances* | | |
| 3. Did all patients get the diagnostic test and reference standard? | ☐Yes ☐ No ☐ Can’t Tell  **X** | |
| *CONSIDER:*   - *were both received regardless of the results of the test of interest* - *Check the 2x2 table (verification bias)* | | |
| 4. Could the results of the test have been influenced by the results of the reference standard? | ☐Yes ☐ No ☐ Can’t Tell  **X** | |
| *CONSIDER:*   - *was there blinding* - *were the tests performed independently* - *could there have been review bias* | | |
| 5. Is the disease status of the tested population clearly described? | ☐Yes ☐ No ☐ Can’t Tell  **X** | |
| *CONSIDER:*   - *presenting symptoms* - *disease stage of severity* - *co-morbidity* - *differential diagnoses (spectrum bias)* | | |
| 6. Were the methods for performing the test described in sufficient detail? | ☐Yes ☐ No ☐ Can’t Tell  **X** | |
| *CONSIDER:*   - *Was a protocol followed* | | |
| **Section B: What are the results?** | | |
| 7. What are the results? | | ☐Yes ☐ No ☐ Can’t Tell  **X** |
| *CONSIDER:*   - *are the sensitivity and specificity and/or likelihood ratios presented* - *are the results presented in such a way that we can work them out* | | |
| 8. How sure are we about the results? Consequences and cost of alternatives performed? | | ☐Yes ☐ No ☐ Can’t Tell  **X** |
| *CONSIDER:*   - *could they have occurred by chance* - *are there confidence limits* - *what are they* | | |
| **Section C: Will the results help locally?** | | |
| 9. Can the results be applied to your patients/the population of interest? | | ☐Yes ☐ No ☐ Can’t Tell  **X** |
| *CONSIDER:*   - *Do you think your patients/population are so different from those in the study that the results cannot be applied, such as age, sex, ethnicity and spectrum bias.* | | |
| 10. Can the test be applied to your patient or population of interest? | ☐Yes ☐ No ☐ Can’t Tell  **X** | |
| *CONSIDER:*   - *resources and opportunity costs* - *level and availability of expertise required to interpret the tests* - *current practice and availability of services* | | |
| 11. Were all outcomes important to the individual or population considered? | ☐Yes ☐ No ☐ Can’t Tell  **X** | |
| *CONSIDER:*   - *will the knowledge of the test result improve patient wellbeing* - *will the knowledge of the test result lead to a change in patient management* | | |
| 12. What would be the impact of using this test on your patients/population?   It supports histology-led diagnosis and highlights risk of false-negative cultures | | |

| **APPRAISAL SUMMARY**: *List key points from your critical appraisal that need to be considered when assessing the validity of the results and their usefulness in decision-making.* | | |
| --- | --- | --- |
| **Positive/Methodologically sound** | **Negative/Relatively poor methodology** | **Unknowns** |
| **Diagnostic design**  **Clear outcomes**  **Good applicability** | **No blinding**  **Culture limitations** | **Precision** |

CASP Checklist:

For diagnostic test studies

| **Reviewer Name:** | Levi Kadiri, Anthony Maher |
| --- | --- |
| **Paper Title:** | Are we misdiagnosing diabetic foot osteomyelitis? Is the gold standard gold? |
| **Author:** | Larvey et al |
| **Web Link:** | Doi : <https://doi.org/10.1053/j.jfas.2018.12.010> |
| **Appraisal Date:** | 09/01/2026 |

During critical appraisal, never make assumptions about what the researchers have done. If it is not possible to tell, use the “Can’t tell” response box. If you can’t tell, at best it means the researchers have not been explicit or transparent, but at worst it could mean the researchers have not undertaken a particular task or process. Once you’ve finished the critical appraisal, if there are a large number of “Can’t tell” responses, consider whether the findings of the study are trustworthy and interpret the results with caution.

| **Section A: Are the results of the study valid?** | | |
| --- | --- | --- |
| 1.Did the study address a clearly formulated research question? | ☐Yes ☐ No ☐ Can’t Tell  **X** | |
| *CONSIDER:*  *A question should include information about*   - *the population* - *the test* - *the setting* - *the outcomes* | | |
| 2.Was there a comparison with an appropriate reference standard? | ☐Yes ☐ No ☐ Can’t Tell  **X** | |
| *CONSIDER:*   - *Is this reference test(s) the best available indicator in the circumstances* | | |
| 3.Did all patients get the diagnostic test and reference standard? | ☐Yes ☐ No ☐ Can’t Tell  **X** | |
| *CONSIDER:*   - *were both received regardless of the results of the test of interest* - *Check the 2x2 table (verification bias)* | | |
| 4.Could the results of the test have been influenced by the results of the reference standard? | ☐Yes ☐ No ☐ Can’t Tell  **X** | |
| *CONSIDER:*   - *was there blinding* - *were the tests performed independently* - *could there have been review bias* | | |
| 5.Is the disease status of the tested population clearly described? | ☐Yes ☐ No ☐ Can’t Tell  **X** | |
| *CONSIDER:*   - *presenting symptoms* - *disease stage of severity* - *co-morbidity* - *differential diagnoses (spectrum bias)* | | |
| 6.Were the methods for performing the test described in sufficient detail? | ☐Yes ☐ No ☐ Can’t Tell  **X** | |
| *CONSIDER:*   - *Was a protocol followed* | | |
| **Section B: What are the results?** | | |
| 7.What are the results? | | ☐Yes ☐ No ☐ Can’t Tell  **X** |
| *CONSIDER:*   - *are the sensitivity and specificity and/or likelihood ratios presented* - *are the results presented in such a way that we can work them out* | | |
| 8.How sure are we about the results? Consequences and cost of alternatives performed? | | ☐Yes ☐ No ☐ Can’t Tell  **X** |
| *CONSIDER:*   - *could they have occurred by chance* - *are there confidence limits* - *what are they* | | |
| **Section C: Will the results help locally?** | | |
| 9 .Can the results be applied to your patients/the population of interest? | | ☐Yes ☐ No ☐ Can’t Tell  **X** |
| *CONSIDER:*   - *Do you think your patients/population are so different from those in the study that the results cannot be applied, such as age, sex, ethnicity and spectrum bias.* | | |
| 10.Can the test be applied to your patient or population of interest? | ☐Yes ☐ No ☐ Can’t Tell  **X** | |
| *CONSIDER:*   - *resources and opportunity costs* - *level and availability of expertise required to interpret the tests* - *current practice and availability of services* | | |
| 11.Were all outcomes important to the individual or population considered? | ☐Yes ☐ No ☐ Can’t Tell  **X** | |
| *CONSIDER:*   - *will the knowledge of the test result improve patient wellbeing* - *will the knowledge of the test result lead to a change in patient management* | | |
| 12.What would be the impact of using this test on your patients/population?  **It reinforces limitations of microbiology alone and suggests adjunctive role for molecular diagnostics** | | |

| **APPRAISAL SUMMARY**: *List key points from your critical appraisal that need to be considered when assessing the validity of the results and their usefulness in decision-making.* | | |
| --- | --- | --- |
| **Positive/Methodologically sound** | **Negative/Relatively poor methodology** | **Unknowns** |
| **Prospective design**  **Clear reference**  **Real diagnostic setting** | **Small sample** | **Blinding**  **Precision** |

CASP Checklist:

For diagnostic test studies

| **Reviewer Name:** | Levi Kadiri, Anthony Maher |
| --- | --- |
| **Paper Title:** | Histopathology is More Reliable Than Microbiology for Detecting Residual Osteomyelitis After Conservative Surgery for Diabetic Foot: The Pitfall of False-Positive Cultures and the Role of Pseudomonas aeruginosa |
| **Author:** | Víquez-Molina et al |
| **Web Link:** | Doi: <https://doi.org/10.1177/15347346251338689> |
| **Appraisal Date:** | 09/01/2026 |

During critical appraisal, never make assumptions about what the researchers have done. If it is not possible to tell, use the “Can’t tell” response box. If you can’t tell, at best it means the researchers have not been explicit or transparent, but at worst it could mean the researchers have not undertaken a particular task or process. Once you’ve finished the critical appraisal, if there are a large number of “Can’t tell” responses, consider whether the findings of the study are trustworthy and interpret the results with caution.

| **Section A: Are the results of the study valid?** | | |
| --- | --- | --- |
| 1.Did the study address a clearly formulated research question? | ☒Yes ☐ No ☐ Can’t Tell | |
| *CONSIDER:*  *A question should include information about*   - *the population* - *the test* - *the setting* - *the outcomes* | | |
| 2.Was there a comparison with an appropriate reference standard? | ☒Yes ☐ No ☐ Can’t Tell | |
| *CONSIDER:*   - *Is this reference test(s) the best available indicator in the circumstances* | | |
| 3.Did all patients get the diagnostic test and reference standard? | ☒Yes ☐ No ☐ Can’t Tell | |
| *CONSIDER:*   - *were both received regardless of the results of the test of interest* - *Check the 2x2 table (verification bias)* | | |
| 4.Could the results of the test have been influenced by the results of the reference standard? | ☐Yes ☐ No ☒ Can’t Tell | |
| *CONSIDER:*   - *was there blinding* - *were the tests performed independently* - *could there have been review bias* | | |
| 5.Is the disease status of the tested population clearly described? | ☒Yes ☐ No ☐ Can’t Tell | |
| *CONSIDER:*   - *presenting symptoms* - *disease stage of severity* - *co-morbidity* - *differential diagnoses (spectrum bias)* | | |
| 6.Were the methods for performing the test described in sufficient detail? | ☒Yes ☐ No ☐ Can’t Tell | |
| *CONSIDER:*   - *Was a protocol followed* | | |
| **Section B: What are the results?** | | |
| 7.What are the results? | | ☒Yes ☐ No ☐ Can’t Tell |
| *CONSIDER:*   - *are the sensitivity and specificity and/or likelihood ratios presented* - *are the results presented in such a way that we can work them out* | | |
| 8.How sure are we about the results? Consequences and cost of alternatives performed? | | ☐Yes ☐ No ☒ Can’t Tell |
| *CONSIDER:*   - *could they have occurred by chance* - *are there confidence limits* - *what are they* | | |
| **Section C: Will the results help locally?** | | |
| 9. Can the results be applied to your patients/the population of interest? | | ☒Yes ☐ No ☐ Can’t Tell |
| *CONSIDER:*   - *Do you think your patients/population are so different from those in the study that the results cannot be applied, such as age, sex, ethnicity and spectrum bias.* | | |
| 10. Can the test be applied to your patient or population of interest? | ☒Yes ☐ No ☐ Can’t Tell | |
| *CONSIDER:*   - *resources and opportunity costs* - *level and availability of expertise required to interpret the tests* - *current practice and availability of services* | | |
| 11. Were all outcomes important to the individual or population considered? | ☒Yes ☐ No ☐ Can’t Tell | |
| *CONSIDER:*   - *will the knowledge of the test result improve patient wellbeing* - *will the knowledge of the test result lead to a change in patient management* | | |
| 12. What would be the impact of using this test on your patients/population?   It supports greater reliance on histopathology and highlights risk of overtreatment if relying on microbiology alone | | |

| **APPRAISAL SUMMARY**: *List key points from your critical appraisal that need to be considered when assessing the validity of the results and their usefulness in decision-making.* | | |
| --- | --- | --- |
| **Positive/Methodologically sound** | **Negative/Relatively poor methodology** | **Unknowns** |
| **Paired testing**  **Clear outcomes**  **Clinically relevant** | **Retrospective design**  **Limited disease spectrum** | **Blinding** **Precision** |

*CASP Checklist:*

*For case control studies*

| ***Reviewer Name:*** | Levi Kadiri, Anthony Maher |
| --- | --- |
| ***Paper Title:*** | Histology versus microbiology for accuracy in identification of osteomyelitis in the diabetic foot. |
| ***Author:*** | Weiner et al |
| ***Web Link:*** | Doi: <https://doi.org/10.1053/j.jfas.2010.12.001> |
| ***Appraisal Date:*** | 10/01/2026 |

*During critical appraisal, never make assumptions about what the researchers have done. If it is not possible to tell, use the “Can’t tell” response box. If you can’t tell, at best it means the researchers have not been explicit or transparent, but at worst it could mean the researchers have not undertaken a particular task or process. Once you’ve finished the critical appraisal, if there are a large number of “Can’t tell” responses, consider whether the findings of the study are trustworthy and interpret the results with caution.*

| ***Section A: Are the results of the study valid?*** | | |
| --- | --- | --- |
| 1. *Did the study address a clearly focused issue?* | *☒Yes ☐ No ☐ Can’t Tell* | |
| *CONSIDER:*  *An issue can be ‘focused’ In terms of*   - *the population studied* - *whether the study tried to detect a beneficial or harmful effect* - *the risk factors studied* | | |
| 2. *Did the authors use an appropriate method to answer their question?* | *☒Yes ☐ No ☐ Can’t Tell* | |
| *CONSIDER:*   - *is a case control study an appropriate way of answering the question under the circumstances* - *did it address the study question* | | |
| 3. *Were the cases recruited in an acceptable way?* | *☒Yes ☐ No ☐ Can’t Tell* | |
| *CONSIDER:*  *We are looking for selection bias which might compromise validity of the findings*   - *are the cases defined precisely* - *were the cases representative of a defined population (geographically and/or temporally)* - *was there an established reliable system for selecting all the cases* - *are they incident or prevalent* - *is there something special about the cases* - *is the time frame of the study relevant to disease/exposure* - *was there a sufficient number of cases selected* - *was there a power calculation* | | |
| 4. *Were the controls selected in an acceptable way?* | *☒Yes ☐ No ☐ Can’t Tell* | |
| *CONSIDER:*  *We are looking for selection bias which might compromise the generalisability of the findings*   - *were the controls representative of the defined population (geographically and/or temporally)* - *was there something special about the controls* - *was the non-response high, could non-respondents be different in any way* - *are they matched, population based or randomly selected* - *was there a sufficient number of controls selected* | | |
| 5. *Was the exposure accurately measured to minimise bias?* | *☒Yes ☐ No ☐ Can’t Tell* | |
| *CONSIDER:*  *We are looking for measurement, recall or classification bias*   - *was the exposure clearly defined and accurately measured* - *did the authors use subjective or objective measurements* - *do the measures truly reflect what they are supposed to measure (have they been validated)* - *were the measurement methods similar in the cases and controls* - *did the study incorporate blinding where feasible* - *is the temporal relation correct (does the exposure of interest precede the outcome)* | | |
| 6. *a) Aside from the experimental intervention, were the groups treated equally?* | *☒Yes ☐ No ☐ Can’t Tell* | |
| *CONSIDER:*  *List the ones you think might be important, that the author may have missed*   - *genetic* - *environmental* - *socio-economic* | | |
| 6 *b) Have the authors taken account of the potential confounding factors in the design and/or in their analysis?* | *☐Yes ☐ No ☒ Can’t Tell* | |
| *CONSIDER:*   - *restriction in design, and techniques e.g. modelling, stratified-, regression-, or sensitivity analysis to correct, control or adjust for confounding factors* | | |
| ***Section B: What are the results?*** | | |
| 7. *Was the exposure effect large?* | | *☒Yes ☐ No ☐ Can’t Tell* |
| *CONSIDER:*   - *what are the bottom-line results* - *is the analysis appropriate to the design* - *how strong is the association between exposure and outcome (look at the odds ratio)* - *are the results adjusted for confounding, and might confounding still explain the association* - *has adjustment made a big difference to the OR* | | |
| 8. *Was the estimate of the exposure effect precise?* | | *☐Yes ☒ No ☐ Can’t Tell* |
| *CONSIDER:*   - *size of the p-value* - *size of the confidence intervals* - *have the authors considered all the important variables* - *how was the effect of subjects refusing to participate evaluated* | | |
| 9. *Do you believe the results?* | | *☒Yes ☐ No ☐ Can’t Tell* |
| *CONSIDER:*   - *big effect is hard to ignore!* - *can it be due to chance, bias, or confounding* - *are the design and methods of this study sufficiently flawed to make the results unreliable* - *consider Bradford Hills criteria (e.g. time sequence, does-response gradient, strength, biological plausibility)* | | |
| ***Section C: Will the results help locally?*** | | |
| 10. *Can the results be applied to your patients/the population of interest?* | | *☒Yes ☐ No ☐ Can’t Tell* |
| *CONSIDER:*   - *the subjects covered in the study could be sufficiently different from your population to cause concern* - *if your local setting is likely to differ much from that of the study* - *can you quantify the local benefits and harms* | | |
| 11. *Do the results of this study fit with other available evidence?* | *☒Yes ☐ No ☐ Can’t Tell* | |
| *CONSIDER:*   - *all the available evidence from RCT’s Systematic Reviews, Cohort Studies, and Case Control Studies as well, for consistency* | | |

***Remember One observational study rarely provides sufficiently robust evidence to recommend changes to clinical practice or within health policy decision making. However, for certain questions observational studies provide the only evidence. Recommendations from observational studies are always stronger when supported by other evidence.***

| ***APPRAISAL SUMMARY****: List key points from your critical appraisal that need to be considered when assessing the validity of the results and their usefulness in decision-making.* | | |
| --- | --- | --- |
| ***Positive/Methodologically sound*** | ***Negative/Relatively poor methodology*** | ***Unknowns*** |
| ***Clear case–control design***  ***Paired specimen analysis***  ***Objective exposure measurement***  ***Good biological plausibility*** | ***Small sample size***  ***No confidence intervals***  ***No power calculation***  ***Single-centre*** | ***Blinding***  ***Residual confounding*** |

***CASP Checklist:***

***For Cohort Studies***

| ***Reviewer Name:*** | Levi Kadiri, Anthony Maher |
| --- | --- |
| ***Paper Title:*** | Evaluation of routine proximal bone analysis for microbiology and histopathology in diabetes‐related foot infections and osteomyelitis |
| ***Author:*** | Jin et al |
| ***Web Link:*** | Doi: <https://doi.org/10.1111/ans.18805> |
| ***Appraisal Date:*** | 10/01/2026 |

***During critical appraisal, never make assumptions about what the researchers have done. If it is not possible to tell, use the “Can’t tell” response box. If you can’t tell, at best it means the researchers have not been explicit or transparent, but at worst it could mean the researchers have not undertaken a particular task or process. Once you’ve finished the critical appraisal, if there are a large number of “Can’t tell” responses, consider whether the findings of the study are trustworthy and interpret the results with caution.***

| ***Section A: Are the results valid?*** | | |
| --- | --- | --- |
| 1. ***Did the study address a clearly focused issue?*** | ***☒Yes ☐ No ☐ Can’t Tell*** | |
| ***CONSIDER:***  ***A question can be ‘focused’ in terms of***   - ***the population studied*** - ***the risk factors studied*** - ***is it clear whether the study tried to detect a beneficial or harmful effect*** - ***the outcomes considered*** | | |
| 2. ***Was the cohort recruited in an acceptable way?*** | ***☒Yes ☐ No ☐ Can’t Tell*** | |
| ***CONSIDER:***   - ***Look for selection bias which might compromise the generalisability of the findings:*** - ***was the cohort representative of a defined population*** - ***was there something special about the cohort*** - ***was everybody included who should have been*** | | |
| 3. ***Was the exposure accurately measured to minimise bias?*** | ***☒Yes ☐ No ☐ Can’t Tell*** | |
| ***CONSIDER:***  ***Look for measurement or classification bias:***   - ***did they use subjective or objective measurements*** - ***do the measurements truly reflect what you want them to (have they been validated)*** - ***were all the subjects classified into exposure groups using the same procedure*** | | |
| 4. ***Was the outcome accurately measured to minimise bias?*** | ***☒Yes ☐ No ☐ Can’t Tell*** | |
| ***CONSIDER:***  ***Look for measurement or classification bias:***   - ***did they use subjective or objective measurements*** - ***do the measurements truly reflect what you want them to (have they been validated)*** - ***has a reliable system been established for detecting all the cases (for measuring disease occurrence)*** - ***were the measurement methods similar in the different groups*** - ***were the subjects and/or the outcome assessor blinded to exposure (does this matter)*** | | |
| 5. ***(a) Have the authors identified all important confounding factors?*** | ***☐Yes ☐ No ☒ Can’t Tell*** | |
| ***CONSIDER:***   - ***list the ones you think might be important, and ones the author missed*** | | |
| ***b) Have they taken account of the confounding factors in the design and/or analysis?*** | ***☐Yes ☒ No ☐ Can’t Tell*** | |
| ***CONSIDER:***   - ***look for restriction in design, and techniques e.g. modelling, stratified-, regression-, or sensitivity analysis to correct, control or adjust for confounding factors*** | | |
| 6. ***a) Was the follow up of subjects complete enough?*** | | ***☒Yes ☐ No ☐ Can’t Tell*** |
| ***CONSIDER:***   - ***the persons that are lost to follow-up may have different outcomes than those available for assessment*** - ***in an open or dynamic cohort, was there anything special about the outcome of the people leaving, or the exposure of the people entering the cohort*** | | |
| ***b) Was the follow up of subjects long enough?*** | | ***☒Yes ☐ No ☐ Can’t Tell*** |
| ***CONSIDER:***   - ***the good or bad effects should have had long enough to reveal themselves*** | | |
| ***Section B: What are the results?*** | | |
| 7. ***What are the results of this study?*** | ***☒Yes ☐ No ☐ Can’t Tell*** | |
| ***CONSIDER:***   - ***what are the bottom line results*** - ***have they reported the rate or the proportion between the exposed/unexposed, the ratio/rate difference*** - ***how strong is the association between exposure and outcome (RR)*** - ***what is the absolute risk reduction (ARR)*** | | |
| 8. ***How precise are the results?*** | ***☐Yes ☐ No ☒ Can’t Tell*** | |
| ***CONSIDER:***   - ***look for the range of the confidence intervals, if given*** | | |
| 9. ***Do you believe the results?*** | ***☒Yes ☐ No ☐ Can’t Tell*** | |
| ***CONSIDER:***   - ***big effect is hard to ignore*** - ***can it be due to bias, chance or confounding*** - ***are the design and methods of this study sufficiently flawed to make the results unreliable*** - ***Bradford Hills criteria (e.g. time sequence, dose-response gradient, biological plausibility, consistency)*** | | |
| ***Section C: Will the results help locally?*** | | |
| 10. ***Can the results be applied to the local population?*** | ***☒Yes ☐ No ☐ Can’t Tell*** | |
| ***CONSIDER:***   - ***Is a cohort study the appropriate method to answer this question*** - ***If the subjects covered in this study could be sufficiently different from your population to cause concern*** - ***If your local setting is likely to differ much from that of the study*** - ***If you can quantify the local benefits and harms*** | | |
| 11. ***Do the results of this study fit with other available evidence?*** | | ***☒Yes ☐ No ☐ Can’t Tell*** |
|  | | |
| 12. ***What are the implications of this study for practice?*** | | ***☒Yes ☐ No ☐ Can’t Tell*** |
| ***CONSIDER:***   - ***one observational study rarely provides sufficiently robust evidence to recommend changes to clinical practice or within health policy decision making*** - ***for certain questions, observational studies provide the only evidence*** - ***recommendations from observational studies are always stronger when supported by other evidence*** | | |

| ***APPRAISAL SUMMARY: List key points from your critical appraisal that need to be considered when assessing the validity of the results and their usefulness in decision-making.*** | | |
| --- | --- | --- |
| ***Positive/Methodologically sound*** | ***Negative/Relatively poor methodology*** | ***Unknowns*** |
| ***Clear cohort design***  ***Objective outcomes***  ***Real-world surgical relevance***  ***Complete follow-up*** | ***Retrospective***  ***No multivariable analysis***  ***No precision estimates*** | ***Confounding***  ***Blinding*** |

**Referencing recommendation:**

CASP recommends using the Harvard style referencing, which is an author/date method. Sources are cited within the body of your assignment by giving the name of the author(s) followed by the date of publication. All other details about the publication are given in the list of references or bibliography at the end.

Example:

*Critical Appraisal Skills Programme (2024). CASP (insert name of checklist i.e. Diagnostic test Checklist.) [online] Available at: insert URL. Accessed: insert date accessed.*

**Creative Commons**

©CASP this work is licensed under the Creative Commons Attribution – Non-Commercial- Share A like. To view a copy of this licence, visit <https://creativecommons.org/licenses/by-nc-sa/4.0/>

**Need further training on evidence-based decision making?** Our online training courses are helpful for healthcare educational researchers and any other learners who:

- Need to critically appraise and stay abreast of the healthcare research literature as part of their clinical duties.
- Are considering carrying out research & developing their own research projects.
- Make decisions in their role, whether that be policy making or patient facing.

**Benefits of CASP Training:**

- Affordable – courses start from as little as £6
- Professional training – leading experts in critical appraisal training
- Self-directed study – complete each course in your own time
- 12 months access – revisit areas you aren’t sure of and revise
- CPD certification - after each completed module

Scan the QR code below or visit <https://casp-uk.net/critical-appraisal-online-training-courses/> for more information and to start learning more.
