# Supplementary material for: Concordance of Microbiological and Histopathological Examination of Bone Biopsies in Diagnosing Diabetic Foot Osteomyelitis, a Systematic Review
Source: J Foot Ankle Res. 2026 Oct 2;19(4):e70223. doi: 10.1002/jfa2.70223 (PMC13632924; doi:10.1002/jfa2.70223)
Supplement: Supplementary file 3 [file JFA2-19-e70223-s003.docx]

QUADAS-2

**Phase 1: State the review question: Lavery et al. 2019 – *“Are We Misdiagnosing Diabetic Foot Osteomyelitis?”***

*Patients (setting, intended use of index test, presentation, prior testing): Adults with moderate/severe DFI and suspected DFO*

*Index test(s): Conventional bone culture; 16S rRNA sequencing. All three tests done on the same bone specimen (no meaningful delay).*

*Reference standard and target condition: Histopathology (bone biopsy).*

**Phase 2: Draw a flow diagram for the primary study**

**Source population**
Adults ≥21 years with moderate or severe diabetic foot infection and suspicion of DFO (IDSA criteria) seen at a tertiary centre.

**Enrolled**
35 consecutive patients meeting inclusion criteria; exclusion of patients with other acute infections, prior foot osteomyelitis, immunosuppression, malignancy, or dialysis. (Final N = **35**)

**Bone sampling (same episode)**
All 35 had bone samples obtained either: Percutaneous bone biopsy (n = 10), or Intraoperative bone sample during surgery (n = 25).

Each sample sent simultaneously for: Conventional microbiological culture (index test 1), Histopathology (reference standard), 16S rRNA sequencing (index test 2)

**Analysis set:**
All 35 patients included in the analysis for: Culture vs histopathology, 16S rRNA vs histopathology, Combined definitions of osteomyelitis

No dropouts, no missing index or reference standard results

**Phase 3: Risk of bias and applicability judgments**

**DOMAIN 2: INDEX TEST(S)**

**If more than one index test was used, please complete for each test.**

QUADAS-2 is structured so that 4 key domains are each rated in terms of the risk of bias and the concern regarding applicability to the research question (as defined above). Each key domain has a set of signalling questions to help reach the judgments regarding bias and applicability.

| **DOMAIN 1: PATIENT SELECTION**  **A. Risk of Bias** |  |  |
| --- | --- | --- |
| Describe methods of patient selection:  Patients were **consecutively enrolled** adults ≥21 years with moderate/severe diabetic foot infection and suspicion of DFO, meeting IDSA criteria. Key exclusions: immunosuppression, malignancy, dialysis, and alternative infectious sources. No inappropriate exclusions. |  |  |
| - Was a consecutive or random sample of patients enrolled? | | Yes |
| - Was a case-control design avoided? |  | Yes |
| - Did the study avoid inappropriate exclusions? |  | Yes |
| **Could the selection of patients have introduced bias?** | **RISK: LOW** | |
| **B. Concerns regarding applicability** |  |  |
| Describe included patients (prior testing, presentation, intended use of index test and setting)**:**  Population fully reflects adults with suspected DFO. Matches review question. | | |
| **Is there concern that the included patients do not match the review question?** | **CONCERN: LOW** | |

| **A. Risk of Bias** |  |
| --- | --- |
| Describe the index test and how it was conducted and interpreted: Microbiology  performed on bone samples and processed using standard lab procedures. No indication of blinding to histopathology, though tests were performed in separate departments. Thresholds (growth/no growth; sequencing detection) were pre-specified. | |
| - Were the index test results interpreted without   knowledge of the results of the reference standard? | Unclear |
| - If a threshold was used, was it pre-specified? | Yes |
| **Could the conduct or interpretation of the index test**  **have introduced bias?** | **RISK: UNCLEAR** |
| **B. Concerns regarding applicability** |  |
| **Is there concern that the index test, its conduct, or interpretation differ from the review question?** | **CONCERN: LOW** |

| **DOMAIN 3: REFERENCE STANDARD**  **A. Risk of Bias** |  |  |
| --- | --- | --- |
| Describe the reference standard and how it was conducted and interpreted:  Histopathology is accepted gold standard for DFO. Blinding to microbiology not clearly reported. | | |
| - Is the reference standard likely to correctly classify the target condition? | | Yes |
| - Were the reference standard results interpreted without knowledge of the results of the index test? | | Unclear |
| **Could the reference standard, its conduct, or its**  **interpretation have introduced bias?** | **RISK: UNCLEAR** | |
| **B. Concerns regarding applicability** |  |  |
| **Is there concern that the target condition as defined by the reference standard does not match the review question?** | **CONCERN: LOW** | |

| **DOMAIN 4: FLOW AND TIMING**  **A. Risk of Bias** |  |  |
| --- | --- | --- |
| Describe any patients who did not receive the index test(s) and/or reference standard or who were excluded from the 2x2 table (refer to flow diagram):  All 35 patients received all tests on the same bone sample. No exclusions.  Describe the time interval and any interventions between index test(s) and reference standard: | | |
| - Was there an appropriate interval between index test(s) and reference standard? | | Yes |
| - Did all patients receive a reference standard? |  | Yes |
| - Did patients receive the same reference standard? |  | Yes |
| - Were all patients included in the analysis? |  | Yes |
| **Could the patient flow have introduced bias?** | **RISK: LOW** | |

QUADAS-2

**Phase 1: State the review question: Tardáguila-García et al. 2021 – *“Diagnostic Accuracy of Bone Culture Versus Biopsy in Diabetic Foot Osteomyelitis”***

*Patients (setting, intended use of index test, presentation, prior testing): Patients with clinical suspicion of DFO (acute or chronic).*

*Index test(s): Bone culture (microbiology). Two bone samples taken at same sitting.*

*Reference standard and target condition: Bone biopsy histology.*

**Phase 2: Draw a flow diagram for the primary study**

**Source population**
Patients with acute or chronic diabetic foot osteomyelitis suspected clinically in a specialist diabetic foot unit.

**Included in study**
52 patients with suspected DFO, fulfilling inclusion criteria. (Final N = **52**)

**Bone sampling procedure (single encounter)**
For each patient, **two bone samples** were obtained at the same time: Sample A → microbiological bone culture (index test)

Sample B → histopathology (reference standard)

**Analysis set**

All 52 patients had interpretable culture and histology results.

Diagnostic accuracy of culture calculated against histology (Se, Sp, PPV, NPV, LR).

No described loss to follow-up or missing test results

**Phase 3: Risk of bias and applicability judgments**

**DOMAIN 2: INDEX TEST(S)**

**If more than one index test was used, please complete for each test.**

QUADAS-2 is structured so that 4 key domains are each rated in terms of the risk of bias and the concern regarding applicability to the research question (as defined above). Each key domain has a set of signalling questions to help reach the judgments regarding bias and applicability.

| **DOMAIN 1: PATIENT SELECTION**  **A. Risk of Bias** |  |  |
| --- | --- | --- |
| Describe methods of patient selection:  Patients with clinically suspected DFO were consecutively included in a specialist centre. No inappropriate exclusions. |  |  |
| - Was a consecutive or random sample of patients enrolled? | | Yes |
| - Was a case-control design avoided? |  | Yes |
| - Did the study avoid inappropriate exclusions? |  | Yes |
| **Could the selection of patients have introduced bias?** | **RISK: LOW** | |
| **B. Concerns regarding applicability** |  |  |
| Describe included patients (prior testing, presentation, intended use of index test and setting)**:**  Population fully reflects adults with suspected DFO, matches review question. | | |
| **Is there concern that the included patients do not match the review question?** | **CONCERN: LOW** | |

| **A. Risk of Bias** |  |
| --- | --- |
| Describe the index test and how it was conducted and interpreted:  Microbiology bone culture | |
| - Were the index test results interpreted without   knowledge of the results of the reference standard? | Unclear |
| - If a threshold was used, was it pre-specified? | Yes |
| **Could the conduct or interpretation of the index test**  **have introduced bias?** | **RISK: UNCLEAR** |
| **B. Concerns regarding applicability** |  |
| **Is there concern that the index test, its conduct, or interpretation differ from the review question?** | **CONCERN: LOW** |

| **DOMAIN 3: REFERENCE STANDARD**  **A. Risk of Bias** |  |  |
| --- | --- | --- |
| Describe the reference standard and how it was conducted and interpreted:  Bone sample histology | | |
| - Is the reference standard likely to correctly classify the target condition? | | Yes |
| - Were the reference standard results interpreted without knowledge of the results of the index test? | | Unclear |
| **Could the reference standard, its conduct, or its**  **interpretation have introduced bias?** | **RISK: UNCLEAR** | |
| **B. Concerns regarding applicability** |  |  |
| **Is there concern that the target condition as defined by the reference standard does not match the review question?** | **CONCERN: LOW** | |

| **DOMAIN 4: FLOW AND TIMING**  **A. Risk of Bias** |  |  |
| --- | --- | --- |
| Describe any patients who did not receive the index test(s) and/or reference standard or who were excluded from the 2x2 table (refer to flow diagram):  All patients received all tests. No exclusion  Both tests were taken from the same encounter; no delays.  All 52 patients had both tests and were analysed.  Describe the time interval and any interventions between index test(s) and reference standard: | | |
| - Was there an appropriate interval between index test(s) and reference standard? | | Yes |
| - Did all patients receive a reference standard? |  | Yes |
| - Did patients receive the same reference standard? |  | Yes |
| - Were all patients included in the analysis? |  | Yes |
| **Could the patient flow have introduced bias?** | **RISK: LOW** | |

QUADAS-2

**Phase 1: State the review question: Weiner et al. 2011 – *“Histology versus microbiology for accuracy in identification of osteomyelitis in the diabetic foot***

*Patients (setting, intended use of index test, presentation, prior testing): Consecutive diabetic patients undergoing surgery for foot infection with suspected osteomyelitis at a tertiary hospital.*

*Index test(s): Microbiology (bone culture). Each bone specimen hemisected and processed in parallel.*

*Reference standard and target condition: Histology (bone biopsy).*

**Phase 2: Draw a flow diagram for the primary study**

**Source population**
Consecutive diabetic patients surgically treated for pedal infection with suspected osteomyelitis in a tertiary care hospital.

**Bone specimens included**
Bone samples obtained intra-operatively from these patients.

Final sample set: **44 bone specimens** analysed.

**Specimen handling (single episode)**
Each bone specimen was **hemisected**:

Half → microbiological culture (index test)

Half → histopathologic examination (reference standard)

**Analysis set**
All 44 specimens had paired culture and histology results.

Disagreement between tests assessed using McNemar’s test.

No partial verification; no reported exclusions after sampling.

**Phase 3: Risk of bias and applicability judgments**

**DOMAIN 2: INDEX TEST(S)**

**If more than one index test was used, please complete for each test.**

QUADAS-2 is structured so that 4 key domains are each rated in terms of the risk of bias and the concern regarding applicability to the research question (as defined above). Each key domain has a set of signalling questions to help reach the judgments regarding bias and applicability.

| **DOMAIN 1: PATIENT SELECTION**  **A. Risk of Bias** |  |  |
| --- | --- | --- |
| Describe methods of patient selection:  Consecutive surgical patients with suspected pedal osteomyelitis, based on intraoperative sampling. No case control design |  |  |
| - Was a consecutive or random sample of patients enrolled? | | Yes |
| - Was a case-control design avoided? |  | Yes |
| - Did the study avoid inappropriate exclusions? |  | Yes |
| **Could the selection of patients have introduced bias?** | **RISK: LOW** | |
| **B. Concerns regarding applicability** |  |  |
| Describe included patients (prior testing, presentation, intended use of index test and setting)**:**  Matches the population of interest (patients with suspected DFO) | | |
| **Is there concern that the included patients do not match the review question?** | **CONCERN: LOW** | |

| **A. Risk of Bias** |  |
| --- | --- |
| Describe the index test and how it was conducted and interpreted:  Bolne microbiology | |
| - Were the index test results interpreted without   knowledge of the results of the reference standard? | Unclear |
| - If a threshold was used, was it pre-specified? | Yes |
| **Could the conduct or interpretation of the index test**  **have introduced bias?** | **RISK: UNCLEAR** |
| **B. Concerns regarding applicability** |  |
| **Is there concern that the index test, its conduct, or interpretation differ from the review question?** | **CONCERN: LOW** |

| **DOMAIN 3: REFERENCE STANDARD**  **A. Risk of Bias** |  |  |
| --- | --- | --- |
| Describe the reference standard and how it was conducted and interpreted:  Bone histology | | |
| - Is the reference standard likely to correctly classify the target condition? | | Yes |
| - Were the reference standard results interpreted without knowledge of the results of the index test? | | Unclear |
| **Could the reference standard, its conduct, or its**  **interpretation have introduced bias?** | **RISK: UNCLEAR** | |
| **B. Concerns regarding applicability** |  |  |
| **Is there concern that the target condition as defined by the reference standard does not match the review question?** | **CONCERN: LOW** | |

| **DOMAIN 4: FLOW AND TIMING**  **A. Risk of Bias** |  |  |
| --- | --- | --- |
| Describe any patients who did not receive the index test(s) and/or reference standard or who were excluded from the 2x2 table (refer to flow diagram):  Bone samples were hemisected immediately and processed in parallel. No missing data  Describe the time interval and any interventions between index test(s) and reference standard: | | |
| - Was there an appropriate interval between index test(s) and reference standard? | | Yes |
| - Did all patients receive a reference standard? |  | Yes |
| - Did patients receive the same reference standard? |  | Yes |
| - Were all patients included in the analysis? |  | Yes |
| **Could the patient flow have introduced bias?** | **RISK: LOW** | |

QUADAS-2

**Phase 1: State the review question: Jin et al. 2024 – *“Evaluation of routine proximal bone analysis for microbiology and histopathology in diabetes-related foot infections and osteomyelitis”***

*Patients (setting, intended use of index test, presentation, prior testing): Patients with diabetes undergoing* ***minor amputations*** *for DFO or DFI at a tertiary centre (Canberra).*

*Index test(s): Proximal bone* ***microbiology*** *(margin culture). Each bone specimen hemisected and processed in parallel.*

*Reference standard and target condition: Histology (bone biopsy).*

**Phase 2: Draw a flow diagram for the primary study**

**Source population**
Adult patients with diabetes mellitus undergoing **minor amputation** for diabetes-related foot infection or osteomyelitis at a tertiary hospital (June–Dec 2020).

**Recruited**
84 patients (77 operations for DFO, 17 for DFI). (N = 84)

**Proximal bone margin sampling (intraoperative, same episode)**
For each operation, proximal bone margin was sampled and sent for: Microbiology (proximal bone culture – index test),

Histopathology (proximal bone histology – reference standard)

**Follow-up and outcomes**

Residual DFO defined primarily by positive proximal histology.

Concordance between microbiology and histology assessed.

Revision operations at 6-months linked back to proximal margin status.

All 84 operations had both margin tests reported; no significant missing data described for index or reference tests.

**Phase 3: Risk of bias and applicability judgments**

**DOMAIN 2: INDEX TEST(S)**

**If more than one index test was used, please complete for each test.**

QUADAS-2 is structured so that 4 key domains are each rated in terms of the risk of bias and the concern regarding applicability to the research question (as defined above). Each key domain has a set of signalling questions to help reach the judgments regarding bias and applicability.

| **DOMAIN 1: PATIENT SELECTION**  **A. Risk of Bias** |  |  |
| --- | --- | --- |
| Describe methods of patient selection: Retrospective series of all diabetic patients undergoing minor amputation in a defined time window. No inappropriate exclusions. |  |  |
| - Was a consecutive or random sample of patients enrolled? | | Yes |
| - Was a case-control design avoided? |  | Yes |
| - Did the study avoid inappropriate exclusions? |  | Yes |
| **Could the selection of patients have introduced bias?** | **RISK: LOW** | |
| **B. Concerns regarding applicability** |  |  |
| Describe included patients (prior testing, presentation, intended use of index test and setting)**:**  The population undergoing minor amputation is slightly narrower than all suspected DFO, but still appropriate for residual osteomyelitis assessment**.** | | |
| **Is there concern that the included patients do not match the review question?** | **CONCERN: LOW** | |

| **A. Risk of Bias** |  |
| --- | --- |
| Describe the index test and how it was conducted and interpreted:  Proximal bone margin culture | |
| - Were the index test results interpreted without   knowledge of the results of the reference standard? | Unclear |
| - If a threshold was used, was it pre-specified? | Yes |
| **Could the conduct or interpretation of the index test**  **have introduced bias?** | **RISK: UNCLEAR** |
| **B. Concerns regarding applicability** |  |
| **Is there concern that the index test, its conduct, or interpretation differ from the review question?** | **CONCERN: LOW** |

| **DOMAIN 3: REFERENCE STANDARD**  **A. Risk of Bias** |  |  |
| --- | --- | --- |
| Describe the reference standard and how it was conducted and interpreted:  Margin histopathology | | |
| - Is the reference standard likely to correctly classify the target condition? | | Yes |
| - Were the reference standard results interpreted without knowledge of the results of the index test? | | Unclear |
| **Could the reference standard, its conduct, or its**  **interpretation have introduced bias?** | **RISK: UNCLEAR** | |
| **B. Concerns regarding applicability** |  |  |
| **Is there concern that the target condition as defined by the reference standard does not match the review question?** | **CONCERN: LOW** | |

| **DOMAIN 4: FLOW AND TIMING**  **A. Risk of Bias** |  |  |
| --- | --- | --- |
| Describe any patients who did not receive the index test(s) and/or reference standard or who were excluded from the 2x2 table (refer to flow diagram):  Same operative specimen used for both tests; no exclusions.  Describe the time interval and any interventions between index test(s) and reference standard: | | |
| - Was there an appropriate interval between index test(s) and reference standard? | | Yes |
| - Did all patients receive a reference standard? |  | Yes |
| - Did patients receive the same reference standard? |  | Yes |
| - Were all patients included in the analysis? |  | Yes |
| **Could the patient flow have introduced bias?** | **RISK: LOW** | |

QUADAS-2

**Phase 1: State the review question: Víquez-Molina et al. 2025 – *“Histopathology is More Reliable Than Microbiology for Detecting Residual Osteomyelitis After Conservative Surgery for Diabetic Foot…”***

*Patients (setting, intended use of index test, presentation, prior testing): Patients undergoing* ***conservative surgery*** *for diabetic foot osteomyelitis in a specialist DFO unit (Costa Rica).*

*Index test(s): Microbiology from bone margin (resection margin cultures). Both taken from proximal resection margin during the same operation.*

*Reference standard and target condition: Histopathology from bone margin (resection margin histology).*

**Phase 2: Draw a flow diagram for the primary study**

**Source population**
Patients with diabetic foot osteomyelitis undergoing **conservative surgery** (bone-preserving procedures) at a diabetic foot unit.

**Included in study**
93 patients undergoing conservative surgery for DFO. (N = 93)

**Intraoperative bone margin sampling**
From each patient, bone obtained from the proximal resection margin, then divided as follows:

Margin microbiological culture (index test)

Margin histopathology (reference standard)

**Analysis set**

All 93 had both margin culture and histology results. Histology used as gold standard for residual osteomyelitis.

Culture results classified as:

True positive (culture+ / histology+ = 52)

False positive (contamination: culture+ / histology− = 23)

False negative (culture− / histology+ = 10)

True negative (culture− / histology− = 8)

No partial verification; all included patients contributed paired index–reference data.

**Phase 3: Risk of bias and applicability judgments**

**DOMAIN 2: INDEX TEST(S)**

**If more than one index test was used, please complete for each test.**

QUADAS-2 is structured so that 4 key domains are each rated in terms of the risk of bias and the concern regarding applicability to the research question (as defined above). Each key domain has a set of signalling questions to help reach the judgments regarding bias and applicability.

| **DOMAIN 1: PATIENT SELECTION**  **A. Risk of Bias** |  |  |
| --- | --- | --- |
| Describe methods of patient selection:  Included all patients undergoing conservative surgery for DFO. No inappropriate exclusions reported |  |  |
| - Was a consecutive or random sample of patients enrolled? | | Yes |
| - Was a case-control design avoided? |  | Yes |
| - Did the study avoid inappropriate exclusions? |  | Yes |
| **Could the selection of patients have introduced bias?** | **RISK: LOW** | |
| **B. Concerns regarding applicability** |  |  |
| Describe included patients (prior testing, presentation, intended use of index test and setting)**:**  Matches the review question closely (diagnostic assessment of bone margins in DFO). | | |
| **Is there concern that the included patients do not match the review question?** | **CONCERN: LOW** | |

| **A. Risk of Bias** |  |
| --- | --- |
| Describe the index test and how it was conducted and interpreted:  Margin bone microbiology | |
| - Were the index test results interpreted without   knowledge of the results of the reference standard? | Unclear |
| - If a threshold was used, was it pre-specified? | Yes |
| **Could the conduct or interpretation of the index test**  **have introduced bias?** | **RISK: UNCLEAR** |
| **B. Concerns regarding applicability** |  |
| **Is there concern that the index test, its conduct, or interpretation differ from the review question?** | **CONCERN: LOW** |

| **DOMAIN 3: REFERENCE STANDARD**  **A. Risk of Bias** |  |  |
| --- | --- | --- |
| Describe the reference standard and how it was conducted and interpreted:  Margin histopathology | | |
| - Is the reference standard likely to correctly classify the target condition? | | Yes |
| - Were the reference standard results interpreted without knowledge of the results of the index test? | | Unclear |
| **Could the reference standard, its conduct, or its**  **interpretation have introduced bias?** | **RISK: UNCLEAR** | |
| **B. Concerns regarding applicability** |  |  |
| **Is there concern that the target condition as defined by the reference standard does not match the review question?** | **CONCERN: LOW** | |

| **DOMAIN 4: FLOW AND TIMING**  **A. Risk of Bias** |  |  |
| --- | --- | --- |
| Describe any patients who did not receive the index test(s) and/or reference standard or who were excluded from the 2x2 table (refer to flow diagram):  *Same bone margin; all 93 had complete paired results*  Describe the time interval and any interventions between index test(s) and reference standard: | | |
| - Was there an appropriate interval between index test(s) and reference standard? | | Yes |
| - Did all patients receive a reference standard? |  | Yes |
| - Did patients receive the same reference standard? |  | Yes |
| - Were all patients included in the analysis? |  | Yes |
| **Could the patient flow have introduced bias?** | **RISK: LOW** | |
